# Atomic Mechanisms of Timothy Syndrome-Associated Mutations in Calcium Channel Cav1.2

**DOI:** 10.3389/fphys.2019.00335

**Published:** 2019-03-29

**Authors:** Vyacheslav S. Korkosh, Artem M. Kiselev, Evgeny N. Mikhaylov, Anna A. Kostareva, Boris S. Zhorov

**Affiliations:** ^1^Almazov National Medical Research Centre, Saint Petersburg, Russia; ^2^I. M. Sechenov Institute of Evolutionary Physiology and Biochemistry, Russian Academy of Sciences, Saint Petersburg, Russia; ^3^Department of Woman and Child Health, Karolinska Institute, Stockholm, Sweden; ^4^Department of Biochemistry and Biomedical Sciences, McMaster University, Hamilton, ON, Canada

**Keywords:** channel gating, channelopathies, cardiac arrhythmias, voltage-dependent inactivation, state-dependent contacts, LQTS, homology modeling, Monte Carlo energy minimizations

## Abstract

Timothy syndrome (TS) is a very rare multisystem disorder almost exclusively associated with mutations G402S and G406R in helix IS6 of Cav1.2. Recently, mutations R518C/H in helix IIS0 of the voltage sensing domain II (VSD-II) were described as a cause of cardiac-only TS. The three mutations are known to decelerate voltage-dependent inactivation (VDI). Here, we report a case of cardiac-only TS caused by mutation R518C. To explore possible impact of the three mutations on interdomain contacts, we modeled channel Cav1.2 using as templates Class Ia and Class II cryo-EM structures of presumably inactivated channel Cav1.1. In both models, R518 and several other residues in VSD-II donated H-bonds to the IS6-linked α1-interaction domain (AID). We further employed steered Monte Carlo energy minimizations to move helices S4–S5, S5, and S6 from the inactivated-state positions to those seen in the X-ray structures of the open and closed NavAb channel. In the open-state models, positions of AID and VSD-II were similar to those in Cav1.1. In the closed-state models, AID moved along the β subunit (Cavβ) toward the pore axis and shifted AID-bound VSD-II. In all the models R518 retained strong contacts with AID. Our calculations suggest that conformational changes in VSD-II upon its deactivation would shift AID along Cavβ toward the pore axis. The AID-linked IS6 would bend at flexible G402 and G406, facilitating the activation gate closure. Mutations R518C/H weakened the IIS0-AID contacts and would retard the AID shift. Mutations G406R and G402S stabilized the open state and would resist the pore closure. Several Cav1.2 mutations associated with long QT syndromes are consistent with this proposition. Our results provide a mechanistic rationale for the VDI deceleration caused by TS-associated mutations and suggest targets for further studies of calcium channelopathies.

## Introduction

Calcium channels play key roles in cell physiology. Entry of calcium ions through calcium channels triggers various processes, including neurotransmitter release, hormone secretion, gene transcription, excitation–transcription coupling, and memory formation; for a recent review see Zamponi et al. (2015). The functional role of calcium current in cardiac myocytes became recognized at the end of XIX century (Ringer, 1883). Several types of calcium channels are expressed in cardiac myocytes depending on localization (atria, ventricles, Purkinje fibers), developmental stage and species (Brette et al., 2006; Benitah et al., 2010). Among these, the L-type calcium channel Cav1.2 has the central role both for generation of the action potential in working myocytes and for excitation–contraction coupling. Inward calcium current through Cav1.2 contributes for the plateau phase of action potential and, on the level of T-tubes, initiates calcium-induced calcium release from sarcoplasmic reticulum, thus supporting the contraction force (Harvey and Hell, 2013). As a consequence, functional alterations in Cav1.2 channels mainly due to CACNA1C gene mutations lead to cardiac arrhythmic disorders such as atrial fibrillation, long QT syndrome and conduction defects, as well as structural cardiac disorders such as cardiomyopathies and congenital heart defects (Benitah et al., 2010).

Cav1.2 consists of the pore-forming α1 subunit and ancillary subunits Cavβ, Cavα2δ, and Cavγ. The pore-forming α1 subunit, which folds from a single polypeptide chain of four homologous repeats, contains the PD and four VSDs. Each repeat includes six transmembrane helices and extracellular membrane-reentering P-loop with selectivity-filter residues between helices P1 and P2. Helices S1–S4 form transmembrane segments of VSDs, while helices S4–S5, S5, P1–P2, and S6 contribute a quarter to PD. The extracellular third of PD is structurally conserved in the open and closed channels, whereas the cytoplasmic half of PD undergoes significant transformations between the open and closed states. The breakthrough cryo-EM structure of the rabbit Cav1.1 channel resolved large parts of the α1 subunit and ancillary subunits (Wu et al., 2015, 2016), thus opening a possibility of structure-based interpretations of numerous experimental data on physiology, pathophysiology, and pharmacology of calcium channels.

Following membrane depolarization, the Cav1.2 channels open and then undergo VDI and CDI, respectively, which are the key mechanisms of the negative feedback regulation. Hypotheses on the VSI and CDI mechanisms include the pore block by a hinged lid located in the cytoplasmic linker between repeats I and II (Stotz et al., 2000, 2004; Barrett and Tsien, 2008), collapse of the selectivity filter, and allosteric inhibition of the activation gate, see Tadross et al. (2010) and references therein. The α-helical segment in linker I/II of T-type calcium channels was proposed to serve as a “gating brake” involved in the channel inactivation (Arias et al., 2008). Mutations in linker I/II of Cav1.2 affect VDI and CDI in the presence and absence of Cavβ, supporting the view that the linker serves as a gating brake during inactivation (Almagor et al., 2012). In the cryo-EM structure of the Cav1.1 channel, the IS6-connected helix AID in linker I/II binds between VSD-II and Cavβ (Wu et al., 2016).

Gain-of-function mutations in the cardiac Cav1.2 channels decelerate VDI and CDI, causing inherited cardiac arrhythmia syndromes; for review see Betzenhauser et al. (2015). Among these is type 8 long QT syndrome, or TS, an extremely rare multisystem disorder with prolonged cardiac action potential (Boczek et al., 2015a; Walsh et al., 2018). Only few TS-associated mutations are explored electrophysiologically. Mutations G^406^R and G^402^S in helix IS6 dramatically decelerate or even abolish VDI (Splawski et al., 2004, 2005; Barrett and Tsien, 2008) and also impair CDI (Dick et al., 2016). Mutations R^518^C/H in helix IIS0 were described in patients with so-called cardiac-only TS including only cardiac abnormalities such as prolongation of QT interval, conduction disorders, cardiomyopathies and ventricular tachycardia (Boczek et al., 2015b). These mutations were demonstrated to have a complex electrophysiological phenotype, including decrease in cell surface expression and inactivation in combination with increased window and late current (Boczek et al., 2015b). The disturbances were much larger when perfusion was done with 15 mM barium rather than with 15 mM calcium, but VDI was significantly decelerated in experiments with the high concentrations of both non-physiological and physiological charge carriers (Boczek et al., 2015b). Atomic mechanisms by which the three TS-associated mutations located in different domains of the channel decelerate VDI are largely unknown.

In this study we describe a familial case of cardiac-only TS presenting with complex clinical phenotype including prolongation of QT interval, atrial fibrillation, congenital heart disorder, ventricular tachycardia, and cardiac conduction defects caused by mutation R^518^C and sought to explore computationally possible structural consequences of mutations R^518^C/H, G^402^S, and G^406^R.

Many channelopathy-associated mutations in sodium channels are located in VSDs and cytoplasmic half of PD that undergo significant rearrangements upon the channel gating (Huang et al., 2017). Such mutations affect residues, which are likely involved in intersegment contacts that stabilize or destabilize various states of the channel. To examine state-dependent contacts, 3D structures of the Cav1.2 channel in different functional states are necessary. In lack of experimental 3D structures of the Cav1.2 channel, we used a homology modeling approach. As principal structural templates for the modeling we employed Class Ia and Class II cryo-EM structures of the rabbit Cav1.1 channel (Wu et al., 2016). These structures are proposed to represent potentially inactivated states because the activation gate is closed and all four voltage-sensing domains are in ‘up’ conformations (Wu et al., 2016). Respective inactivated-state models ^i^Cav1.2-I and ^i^Cav1.2-II comprise the α1 and Cavβ subunits. In the Cav1.1 structure, the resolved part of Cavβ forms close contacts with the resolved part of AID and approaches helix IIS0 at the cytoplasmic side of VSD-II. To model the Cav1.2 channel in the open and closed states, we used as templates respective X-ray structures of a bacterial sodium channel NavAb (Lenaeus et al., 2017). Since NavAb lacks AID and Cavβ, the X-ray structures *per se* are insufficient for building full-fledged equivalents of the inactivated-state Cav1.2 models. Therefore, we employed steered Monte Carlo minimizations (MCMs) to move in a stepwise manner the PD helices S4–S5, S5, and S6 in ^i^Cav1.2-I and ^i^Cav1.2-II to positions, which are seen in respective states of NavAb.

The *in silico* opening and closing of PD caused a significant displacement of AID and certain perturbation of VSD-II. We analyzed multiple contacts between these segments in different states. The AID contacts with VSD-II and Cavβ in the open-state models are rather similar to respective inactivated-state models. In contrast, in the closed-state models IS6-linked AID moved along Cavβ and shifted IIS0 and loop IIS2-S3, causing noticeable perturbations of VSD-II.

Our models imply that conformational perturbations within VSD-II, which follow the channel opening, would shift AID along Cavβ toward the pore axis. The AID-linked IS6 would bend at flexible G^402^ and G^406^ and shift toward the pore axis, thus initiating the activation gate closure in the process of VDI. Mutations R^518^C/H would retard the AID shift upon VSD-II perturbation and thus decelerate VDI. Substitutions G^406^R and G^402^S would rigidify IS6, stabilize the open state, and thus also decelerate VDI. Our results provide a mechanistic rationale for the VDI deceleration caused by TS-associated mutations and suggest targets for further mutational analysis.

## Materials and Methods

### Mutation Identification

The study was performed according to the Declaration of Helsinki, and approval was obtained from the Ethical Review Boards of Almazov National Medical Research Centre, approval number 2014/95. Written informed consent was obtained from the study subject, including a consent for publication of the clinical case. Target next generation sequencing was performed on Illumina MiSeq using Haloplex custom target enrichment kit (Agilent; Waldbronn, Germany) with a panel of 108 genes associated with cardiac disorders as earlier described (Kostareva et al., 2016). All disease-related genetic variants were subsequently validated by Sanger sequencing and classified according to American College of Medical Genetics guidelines (Richards et al., 2015).

### Modeling Cav1.2 in Inactivates States

Class Ia and Class II cryo-EM structures, which captured the Cav1.1 channel in presumably inactivated states (Wu et al., 2016), were used as templates to build inactivated-state models ^i^Cav1.2-Ia and ^i^Cav1.2-II. Methodology of our homology modeling approach with the ZMM program is described elsewhere (e.g., Bruhova and Zhorov, 2010; Garden and Zhorov, 2010; Tikhonov and Zhorov, 2017b). Briefly, ZMM minimizes energy in the space of internal (generalized) coordinates. These include torsion angles, positions (Cartesian coordinates) of “root atoms” at the N-ends of segments, which are not covalently bonded to other segments, and orientations (Euler angles) of triplets of atoms at the roots of free segments. Bond lengths and bond angles were kept rigid, except of bond angles of prolines. Aromatic rings were also rigid. The models were optimized by the MCM method (Li and Scheraga, 1987) in the space of generalized coordinates. Non-bonded interactions were calculated using the AMBER force field (Weiner et al., 1984, 1986) with the distance cutoff of 9 Å and a shifting function (Brooks et al., 1985). Electrostatic interactions were calculated using the distance- and environment-dependent dielectric function (Garden and Zhorov, 2010). Interactions involving ionized groups were computed without any cutoff.

Those Cav1.2 residues, which in the sequence alignment mismatch with Cav1.1 residues (Supplementary Figure S1), were assigned all-trans starting conformations. The models were optimized in three consecutive MCM trajectories. In the first trajectory, positions and orientations of segments roots, backbone torsions and proline bond angles were kept rigid, while side chain torsions were flexible. In the second trajectory, all generalized coordinates were flexible and “pin” constraints were imposed to prevent large backbone deformations due to clashes, which were not relaxed in the first trajectory. A pin is a flat-bottom parabolic energy function with the force constraint of 10 kcal mol^-1^Å^-2^ that adds the penalty energy if a model C^α^ atom deviates from the template matching atom by more than *d* Å. Pins with *d* = 1 Å were used by default. The third MCM trajectory was computed without pins to ensure that the model is energetically stable. Each MCM trajectory was terminated when the last 2,000 energy minimizations did not decrease the lowest energy found in the trajectory.

We designate residues of the α1 subunit by their genuine numbers in *h*Cav1.2 (Supplementary Figure S1) and residues in the β-subunit as in the Cav1.1 cryo-EM structure (Supplementary Figure S2). To designate PD residues we also use a nomenclature, which is universal for P-loop channels (Zhorov and Tikhonov, 2004). This nomenclature facilitates recognition of symmetric positions in the four repeat domains (Table 1).

**Table 1:** Sequence alignment of helices in the pore domain of P-loop channels^a,b,c^.

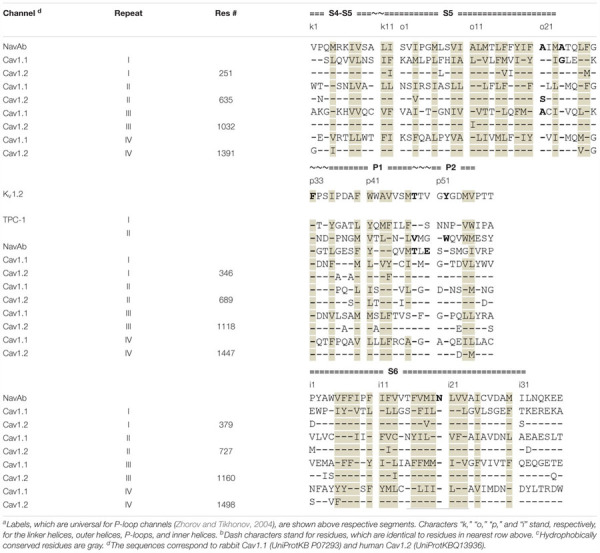

To compare different models, they were superposed by minimizing RMS deviations of alpha carbons in positions p38–p48 of the P1 helices (Table 1), which are the most 3D conserved segments in X-ray and cryo-EM structures of various P-loop channels (Tikhonov and Zhorov, 2017a).

### Transforming Cav1.2 to the Open and Closed States With Steered MCM

The inactivated-state structures do not suggest the direction and magnitude of likely coupled movements of VSD-II, AID and IS6 upon the channel gating. There are two possibilities to model these movements with steered MCM. The first one is to move VSD-II between the activated- and resting-state conformations and monitor displacement of AID and IS6. However, only one structure of a P-loop channel with VSD in the resting (down) position is available; this is a two-pore potassium channel TPC-1 (Guo et al., 2016). In our preliminary steered-MCM computations, the forced movement of VSD-II between the resting and open states shifted AID and AID-linked IS6, but did not significantly perturb other S6 helices. The second and more reliable approach is to move the PD helices S4–S5, S5, and S6 from the inactivated-state positions, which are inherited from the cryo-EM-structures, to the open- and closed-state positions, which are seen in experimental structures of many P-loop channels, and monitor displacements in AID and VSD-II.

Relative disposition of the α1 and β subunits in different states is unknown. Our preliminary attempt to close model ^i^Cav1.2-Ia failed: Cavβ maintained strong contacts with AID, followed its displacement to the pore axis, and lost contacts with VSD-II. At first sight, this is consistent with a significant dislocation of the Cavβ subunit in Class Ia and Class II reconstructions of Cav1.1 (Wu et al., 2016). However, the Cav1.1 cryo-EM structures are obtained without membrane and membrane-anchoring proteins. Therefore, the Cavβ shift between the two reconstructions may be due to strong interactions of Cavβ with the α1 subunit, which are not counterbalanced by interactions with membrane and other proteins. To maintain the general disposition, but permit certain Cavβ mobility, we imposed pins (see section “Modeling Cav1.2 in Inactivates States”) that allowed penalty-free displacement of C^α^ atoms in Cavβ up to 2.5 Å from the cryo-EM templates. In other words, in our models Cavβ served as a soft bedplate for the sliding AID. In lack of experimental data on specific displacements of VSD-I, VSD-III, and VSD-IV upon the channel gating, their C^α^ atoms were constrained with rather flexible pins (*d* = 2 Å) to prevent their possible shifts that would be hardly possible to interpret in physiological terms.

Steered MCM was used to target C^α^ atoms in segments S4–S5, S5, and S6 of all four repeats to matching atoms in the open- and closed-NavAb structures (Table 1). In these calculations no pin constraints were imposed to VSD-II and AID to allow their free movements. Distance constraints, which are analogous to pins (see section “Modeling Cav1.2 in Inactivates States”), were imposed between C^α^ atoms in helices S4–S5 (positions *k3 – k11*), S5 (positions *o1 – o25*), and S6 (*positions i9 – i35*) and matching atoms in the NavAb templates. Additional constraints imposed a limit of 9 Å for deviations of the AID backbone from that in Cavβ.

Distances between some matching C^α^ atoms in the inactivated-state models and the target templates are big. For example, in the superposed structures of ^i^Cav1.2-I and closed-state NavAb, matching C^α^ atoms at the C-end of S6 are 7.2 Å apart. At such distances the pin constraints with the force constant of 10 kcal mol^-1^Å^-2^ (see section “Modeling Cav1.2 in Inactivates States”) induce large forces that would irreversibly damage the models. To prevent this, conformations of segments S4–S5 and S5 were preserved by constraints that imposed alpha-helical H-bonds. Alpha-helical structures of AID (positions *424–447*) and C-terminal parts of S6s (positions *i21 – i36*) were preserved by constraints, which limited to 5° deviations of the model backbone torsions from respective values in the inactivated-sate models.

To ensure small structural changes between two consecutive steps during steered MC minimizations, the number of iterations in each energy minimization was limited to 100. Each point (step), which was accepted to the MCM trajectory, served as the starting point for the next step. Therefore, generalized coordinates, which were insufficiently optimized in the previous step, were further optimized in the next step. In these computations, the unpinned segments and those pinned with wide flat-bottom pins followed the forced displacements of the PD helices. When a target structure in a steered MCM was reached, computations continued until 2,000 consecutive energy minimizations did not decrease the energy. Upon convergence of the steered-MCM trajectory, another MCM trajectory was computed without any constraints to ensure stability of the *in silico* transformed structure. The above protocol was tuned during preliminary calculations to reach a compromise between the model flexibility and integrity.

### Modeling Cav1.2 Mutants

To facilitate computations of mutants R^518^R/C, G^402^S, and G^406^R we used a double-shell approach (Garden and Zhorov, 2010) in which a mutated residue is the center of a flexible inner shell comprising residues that have at least one heavy atom within 15 Å from the mutated residue. The second (outer) shell comprises fixed residues that do not belong to the flexible shell and have at least one heavy atom within 20 Å from the mutated residue. The rigid shell prevents flexible-shell residues to move in the region, which is occupied by residues in the full-fledged model. The starting conformations of the flexible and fixed shells were taken from respective models of WT channel. Generalized coordinates of the mutated residue and residues in the flexible shell were randomly sampled in 128 starting conformations. From each starting point the energy was MC-minimized. The double shell model significantly facilitates computations due to limited number of atoms and variables.

## Results

### Clinical Case

A 48 year old woman presented with atrial fibrillation and no history of arterial hypertension, obesity, endocrine disorders or diastolic cardiac dysfunction. Echocardiography confirmed normal cardiac structure with no signs of hypertrophy or cardiac dilation. Holter monitoring revealed marked prolongation of QT interval (490–560 ms), cardiac conduction defects namely SA and AV block type II and episodes of non-sustained ventricular tachycardia. Familial history reported multiple cases of congenital cardiac defects and sudden cardiac death, as well as Brugada-like ECG pattern in proband’s son. Genetic screening identified mutation R^518^C in CACNA1C gene (NM_001129830: c.C1552T), which according to American College of Medical Genetics classification and ClinVar database is considered to be pathogenic. Due to repeated syncope, a cardioverter-defibrillator was implanted. Currently, all available relatives refuse cascade genetic screening.

### Composition of Cav1.2 Models

**FIGURE 1:**
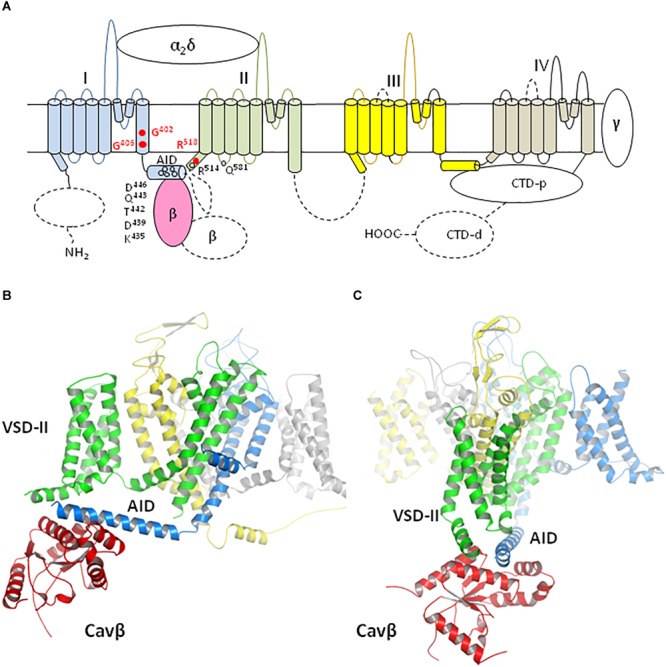
Membrane topology and 3D model of Cav1.2 channel. **(A)** Membrane topology. Dashed lines show those parts of the α1 and Cavβ subunits, which are not resolved in the Cav1.1 cryo-EM stricture and therefore were not modeled. Repeat domains I, II, III, and IV are blue, green, yellow, and gray, respectively. The Cavβ part, which is resolved in the Cav1.1 structure, is light-red. The α2δ and γ subunits and the proximal C-terminal domain (CTD-p), which are resolved in the Cav1.1 structures, were not modeled because in these structures they are far from the residues whose TS-associated mutations are the focus of this study. These residues are shown by red circles. Open circles indicate some of the polar residues, which are involved in H-bonds between AID and VSD-II. **(B,C)** Model ^i^Cav1.2-Ia viewed at different angles. Repeat domains of subunit α1 and subunit β are colored as **(A)**.

The membrane topology and disposition of subunits in the Cav1.1 structure (Wu et al., 2016) are shown in Figure 1A. The sequence alignment of α1 subunits in *h*Cav1.2 and rabbit Cav1.1 channels is given in Supplementary Figure S1 where residues not resolved in the cryo-EM structure are gray. The sequence identity between hCav1.2 and rabbit Cav1.1 (from the N-end of IS0 to the C-end of IVS6) is 70.8%, strongly suggesting similar folding of the channels.

Ancillary subunits α2δ and γ, as well as p-CTD, which are resolved in the cryo-EM structures, are known to modulate functions of Cav1.2 (Hulme et al., 2006; Yang et al., 2011; Dolphin, 2013; Hofmann et al., 2014; Savalli et al., 2016). We did not include Cav-α2δ, Cav-γ and p-CTD in our models (Figure 1B,C) because they are far from residues, which are involved in TS-associated mutations and their contacts, the focus of our study. We did not attempt to model *de novo* those parts of the Cav1.2 complex, whose analogs are not resolved in the Cav1.1 structures (dashed lines in Figure 1A). These include C-terminal part of Cavβ and several fragments of the α1 subunit: N-terminal domain, d-CTD, extracellular loops IIIS3–S4 and IVS3–S4, linker II/III, and C-terminal part of linker I/II. Many mutations in these fragments are associated with calcium channelopathies listed in ClinVar (Landrum et al., 2018), implying importance of respective WT residues for the channel function. The fact that these fragments are not resolved suggests that they adopt different conformations in the protein samples, which were collected to generate the cryo-EM structures. *In vivo*, these fragments may be structured due to interactions with membrane and intracellular proteins. In lack of structural data on such interactions attempts to model these fragments would be premature.

Mutations G^402^S and G^406^R retard inactivation of the Cav1.2 α1 subunit, which was coexpressed with either β2a or β1c subunit (Barrett and Tsien, 2008). In the sequence alignment of human subunits (β2a and β1c with the β subunit, which is partially resolved in the Cav1.1 structure (Supplementary Figure S2), the majority or residues that form contacts with the α1 subunit (Supplementary Table S1) are identical. Exceptions are Eβ258 in β2a vs. Dβ261 in β1c and Dβ306 in Cavβ. Therefore, we have built our models with those Cavβ segments, which are resolved in the Cav1.1 structures.

### Inactivated State Models ^i^Cav1.2-Ia and ^i^Cav1.2-II

Class Ia and Class II cryo-EM structures are proposed to represent “potentially inactivated” states because the activation gate is closed, whereas the voltage-sensing segments S4 are in the “up” conformations (Wu et al., 2016). It should be noted that the S4 segments are in the “up” position practically in all voltage-gated channels whose 3D structures in the open and/or closed states are available, indicating that this position is energetically preferable. The negative membrane potential, which keeps S4s “down” in the resting channels, is absent in crystals or protein samples used to generate cryo-EM structures. Nevertheless, the PD conformation in the Cav1.1 cryo-EM structures is substantially different from the PD conformations seen in the open and closed homotetrameric potassium and sodium channels. Therefore we used the Cav1.1 structures as templates to build models ^i^Cav1.2-Ia and ^i^Cav1.2-II, which, following (Wu et al., 2016), we call inactivated-state models.

**FIGURE 2:**
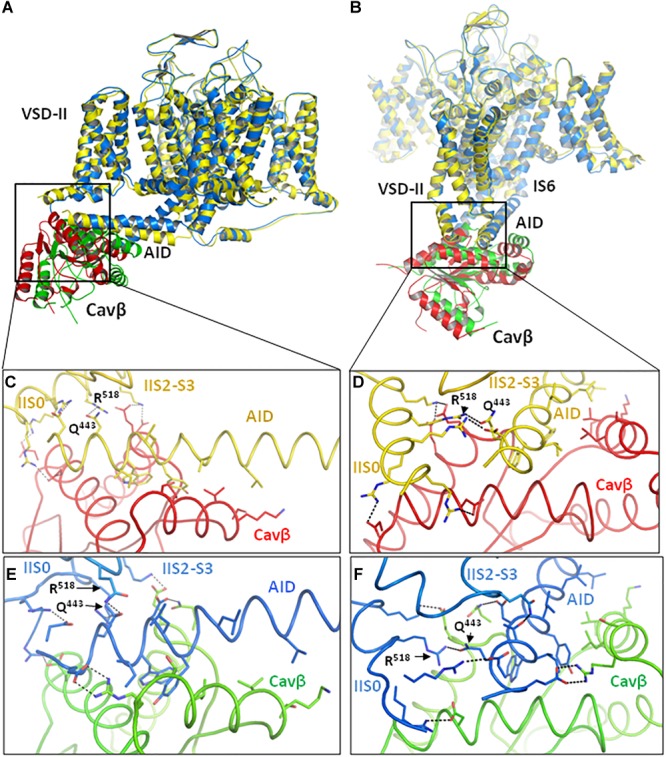
Intersegment contacts are different in two inactivated-state models, ^i^Cav1.2-Ia and ^i^Cav1.2-II. **(A,B)** Superposed models ^i^Cav1.2-Ia (yellow α1 subunit, red Cavβ) and ^i^Cav1.2-II (blue Cav-α1, green Cavβ) viewed at different angles. **(C,D)** Contacts between AID, VSD-II, and Cavβ in model ^i^Cav1.2-Ia. **(E,F)** Contacts between AID, VSD-II, and Cavβ in model ^i^Cav1.2-II. Note significant backbone shifts between the models. Side chains of some residues, which are involved in interdomain contacts (Figure 3 and Supplementary Table S1), are shown as sticks.

In the MC-minimized models ^i^Cav1.2-Ia and ^i^Cav1.2-II all residue clashes that appeared in the starting conformations, were relaxed. RMS deviations of C^α^ atoms in models ^i^Cav1.2-Ia and ^i^Cav1.2-II from matching atoms in the Cav1.1 templates are 0.635 and 0.603 Å, respectively, indicating that the folding of MC-minimized models is very similar to that of respective templates. Superposition of the two models is shown in Figure 2. The PD helices have similar mutual disposition, whereas the positions of AID, Cavβ and cytoplasmic side of VSD-II are different.

**FIGURE 3:**
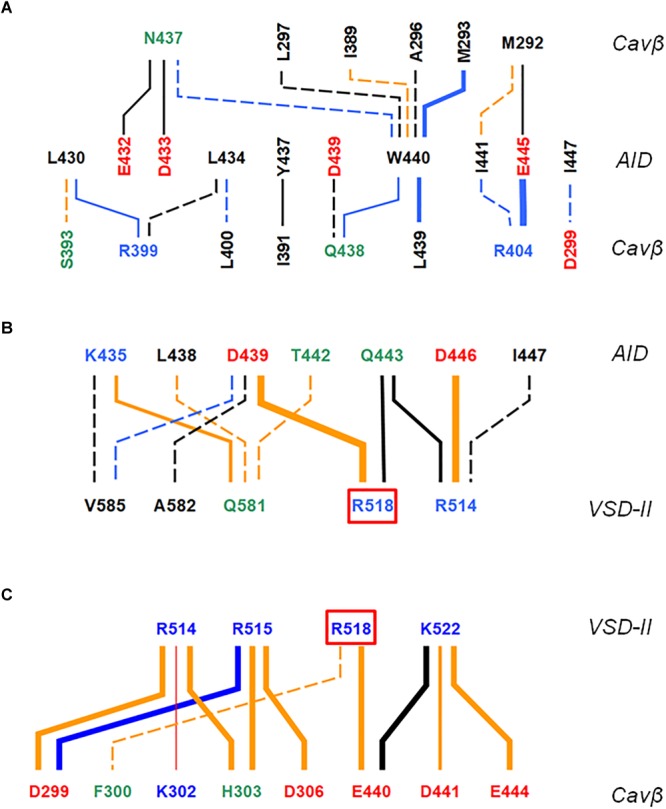
AID contacts with VSD-II and Cavβ in inactivated-state models. Orange, blue and black lines show contacts, which are established, respectively, only in ^i^Cav1.2-Ia, only in ^i^Cav1.2-II, and in both ^i^Cav1.2-Ia, and ^i^Cav1.2-II. Dotted, thin, intermediate and thick lines represent, respectively, contacts with the following energies (kcal/mol): –0.9 to –1.5, –1.51 to –3.0, –3.01 to –5.0, and E < –5.0 (see Supplementary Table S1 for contact energies). Weak contacts with energy –0.4 to –0.9 kcal/mol (Supplementary Table S1) are not shown for clarity. Labels of basic, acidic, polar non-charged and hydrophobic or predominately hydrophobic (Y, W, M) residues are blue, red, green, and black, respectively. **(A)** Contacts AID/Cavβ. **(B)** Contacts AID/VSD-II. **(C)** Contacts VSD-II/Cavβ. A red line shows the only repulsive contacts R^514^: K^β302^ in model ^i^Cav1.2-Ia (Supplementary Table S1).

Contacts between AID, VSD-II and Cavβ are shown in Figure 3 and energies of the contacts are given in Supplementary Tables S1, S2. In both models, VSD-II forms predominantly polar contacts with AID and Cavβ, which in model ^i^Cav1.2-Ia are much stronger than in model ^i^Cav1.2-II (Supplementary Table S1). Four basic residues in IIS0 (R^514^, R^515^, R^518^, and K^522^) provide large contributions to the interaction energy between AID and Cavβ (Figure 3C). In both models, arginine R^518^ forms an H-bond with Q^443^ at the C-end of AID. In model ^i^Cav1.2-Ia, R^518^ also forms salt bridges with D^439^ and E^β440^.

In contrast to mostly polar contacts of AID with VSD-II (Figure 3B), contacts of AID with Cavβ are predominately hydrophobic (Figure 3A) and in model ^i^Cav1.2-II they are usually stronger than in model ^i^Cav1.2-Ia (Supplementary Table S1). Some hydrophobic contacts, in particular those involving aromatic residues Y^437^ and W^440^, are seen in both models (Supplementary Table S1). Tryptophan and tyrosine residues frequently appear in protein-protein contacts (see, e.g., Moreira et al., 2007). Multiple contacts of Y437 and W440 with Cavβ, which are seen in the Cav1.1 structures, are in agreement with the X-ray structures of AID-containing fragments of Cav1.2 with Cavβ (Van Petegem et al., 2004; Almagor et al., 2012).

The enlarged region of contacts between AID, VSD-II, and Cavβ (Figure 2A,B) shows significant shifts between backbones in the superposed models ^i^Cav1.2-Ia and ^i^Cav1.2-II. However, in both models R^518^ maintains its contacts with AID (Figure 2C–F). AID residues 430–446 are located at two helical faces of opposite polarity. The predominately hydrophilic face with R^435^, D^439^, T^442^, Q^443^, and D^446^ forms H-bonds and salt bridges with VSD-II (Figure 3B). The predominantly hydrophobic face with L^430^, L^434^, Y^437^, W^440^, and I^441^ forms hydrophobic contacts with Cavβ (Figure 3A). This feature suggests that AID may work as a sliding stick of the gating brake that transfers conformational perturbations from VSD-II to the cytoplasmic end of IS6, thus initiating the activation gate closure. The polar face of AID would maintain contacts with VSD-II, while predominantly hydrophobic, greasy interface between AID and Cavβ would decrease friction upon the AID sliding. This possibility is explored in Section “Cav1.2 Models in the Open and Closed States.”

Structural analogs of two-faced mobile AID helix can be found in the Cav1.2 models. Thus, the voltage-sensing helix IIS4 has a predominantly hydrophobic face, which interacts with hydrophobic residues in IIIS5 and a predominantly hydrophilic face with basic residues interacting with polar residues in IIS2 and IIS3 (Supplementary Figure S3). The greasy interface IIS4/IIIS5 maintains contacts between VSD-II and PD and minimizes friction upon voltage-dependent movement of IIS4.

### Cav1.2 Models in the Open and Closed States

We have transformed inactivated-state Cav1.2 models in the open (^o^Cav1.2-Ia and ^o^Cav1.2-II) and closed (^c^Cav1.2-Ia and ^c^Cav1.2-II) states as descried Methods (see section “Transforming Cav1.2 to the Open and Closed States With Steered MCM”). Various disease-associated mutations in the C-part of linker I/II are known (Landrum et al., 2018) implying importance of respective residues. However, since this part is not resolved in the Cav1.1 cryo-EM structures (Figure 1A), we did not model it. Proximity of VSD-II to linker I/II and likely impact of the disease-associated mutations on the channel gating suggest that VSD-II interacts with the unresolved C-part of linker I/II as well as with the resolved C-end of AID, which immediately precedes it. To bias contacts between VSD-II and the C-end of AID, we imposed a distance constraint between residues R^518^ and Q^443^ that are H-bonded in both inactivated-state models (Figure 2C–F, 3B and Supplementary Table S1).

**FIGURE 4:**
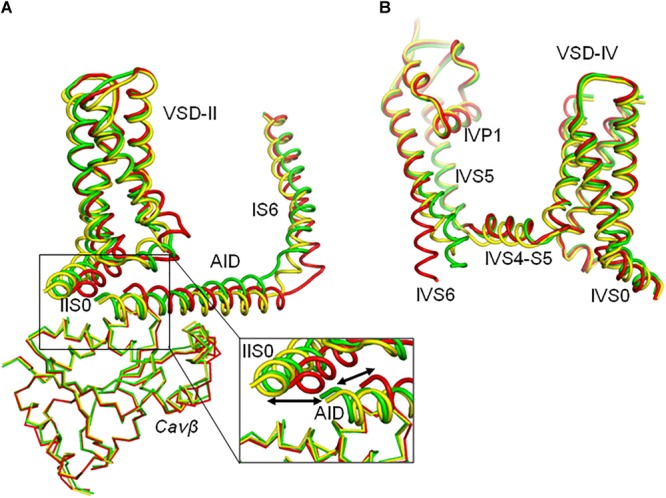
Upon *in silico* closure of the PD, AID slides over Cavβ and shifts IIS0. For clarity, shown are only some parts of models ^i^Cav1.2-Ia (yellow), ^o^Cav1.2-Ia (green), and ^c^Cav1.2-Ia (red). **(A)** Large displacements are seen for AID and VSD-II whose C^α^ atoms were not pinned during steered MCM. The enlarged region shows significant shifts of AID and IIS0 between the open and closed states. Shift of Cavβ is much smaller than the limits of penalty-free deviations of its C^α^ atoms (2.5 Å, see section “Transforming Cav1.2 to the Open and Closed States With Steered MCM”). **(B)** Shift of VSD-IV is much smaller than the limits of penalty-free deviations of its C^α^ atoms (2.0 Å, see section “Transforming Cav1.2 to the Open and Closed States With Steered MCM”). Shifts of VSD-III and VSD-I (these domains are not shown for clarity) are also much smaller that that of VSD-II.

In models ^i^Cav1.2-Ia and ^o^Cav1.2-Ia, positions of VSD-II, AID, and Cavβ are rather similar (Figure 4A). However, positions of the inner helices S6 are different, especially at the their cytoplasmic ends (Figure 4A,B, 5A,B). *In silico* closing the PD in model ^i^Cav1.2-Ia forced the IS6-linked AID to slide over Cavβ and shift VSD-II (Figure 4A). Similar results were obtained for models ^i^Cav1.2-II and ^o^Cav1.2-II (not shown). Since intersegment contacts in the Cav1.1-Ia based models are generally stronger than those in the Cav1.1-II based models (Supplementary Table S2), below we describe manly the former models.

**FIGURE 5:**
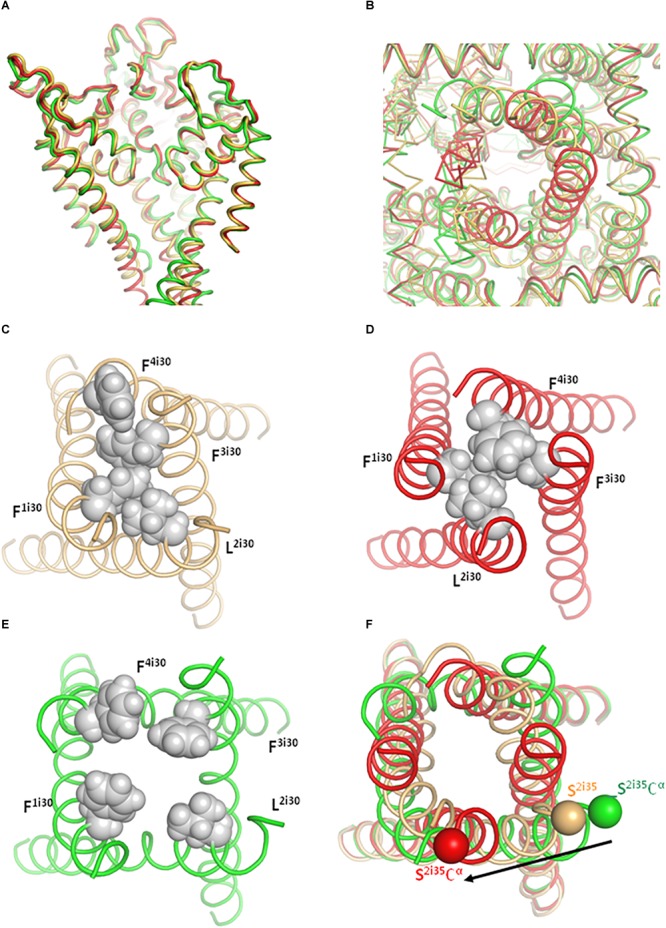
The cytoplasmic part of the PD adopts different conformations in the inactivated (yellow), open (green), and closed (red) models. **(A)** Side view of the PD in the Cav1.1-Ia based models. Note the conserved 3D structure of the extracellular half, but divergent inner helices in the intracellular half. For clarity, cytoplasmic extensions of helices S6 are not shown and the front repeat is removed. **(B)** *C*ytoplasmic view of the PD. One S6 helix is shown as C^α^ tracing and other helices as thin ribbons. **(C–E)** Cytoplasmic views of the S6 bundle. The permeation pathway is tightly sealed by hydrophobic residues in positions i30 in the inactivated **(C)** and closed **(D)** states, but not in the open state **(E)**. **(F)** The C-ends of helices S6 experience significant shifts between the open and closed state. The shift direction and magnitude are shown by an arrow next to positions of an alpha carbon at the C-end of IIS6, which is connected to AID (not shown).

The superposed models ^o^Cav1.2-Ia and ^c^Cav1.2-Ia show significant shifts of backbones in AID, VSD-II, and cytoplasmic half of PD (Figure 4A). However, the backbone shift of Cavβ (Figure 4A) is much smaller than the penalty-free deviation limit of 2.5 Å (see section “Transforming Cav1.2 to the Open and Closed States With Steered MCM”). In other words, AID slid over Cavβ without significantly shifting it. The backbone shifts in VSD-IV (Figure 4B), as well as those in VSD-I and VSD-III (not shown) are also much smaller than the penalty-free deviation limit of 2 Å, which is set for their C^α^ atoms.

In the Cav1.1 structures (Wu et al., 2016) as well as in our inactivated- and closed-state models hydrophobic residues tightly seal the ion-permeation pathway (Figure 5C,D and Supplementary Figure S4). In the open-state models, the hydrophobic residues diverge (Figure 5E). The narrowest level of the permeation pathway at the activation-gate region is formed by hydrophobic residues F^1i30^, L^2i30^, F^3i30^, and F^4i30^ (Figure 5C–E and Table 1). The minimal distances between diagonally opposed atoms at this level are 6.9 Å for residues F^1i30^ and F^3i30^ and 10.5 Å for residues L^2i30^ and F^4i30^. Given flexibility of residue sidechains, mobility of water molecules and certain “breathing” of S6s helices in the open channel, the hydrated calcium ions would pass through the open gate in our open-state model. This property, which is observed in various ion channels, is referred to as “hydrophobic gating” (Aryal et al., 2015).

**FIGURE 6:**
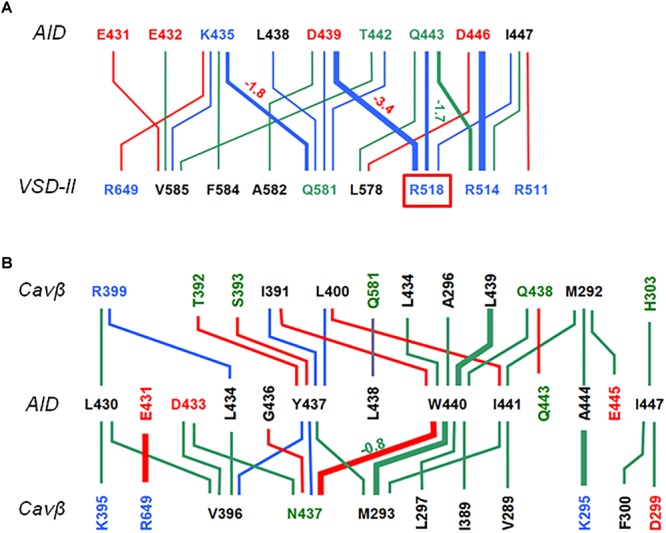
Contacts of AID with VSD-II **(A)** and Cavβ **(B)**. Green, red, and blue lines show, respectively, contacts established only in ^o^Cav1.2-Ia, only in ^c^Cav1.2-Ia, and in both ^o^Cav1.2-Ia and ^c^Cav1.2-Ia. Thin, intermediate and thick lines represent, respectively, contacts with the following energy (kcal/mol): –0.5 to –2.5, –2.51 to –5.0, and E < –5.0. For contacts whose open- and closed-state energies are substantially different, energies of weaker contacts are shown in red for closed state and green for open state. Note that the strongest contacts between AID and VSD are state-independent (thick blue lines), whereas most of contacts between AID and Cavβ are state-dependent (green and read lines).

Multiple intersegment contacts in models ^o^Cav1.2-Ia and ^c^Cav1.2-Ia are illustrated in Figure 6 where red and green lines show contacts established, respectively, in the closed and open states, whereas blue lines show contacts, which are seen in both the open and closed states. The line thickness encodes the contact strength. Contact energies are given in Supplementary Table S2. Strong intersegment contacts (salt bridges and H-bonds) between long sidechains in AID and VSD-II are generally state-independent (thick blue lines in Figure 6A). In contrast, most of contacts between AID and Cavβ are state-dependent (green and red lines in Figure 6B). Below we provide structural interpretation of this finding.

**FIGURE 7:**
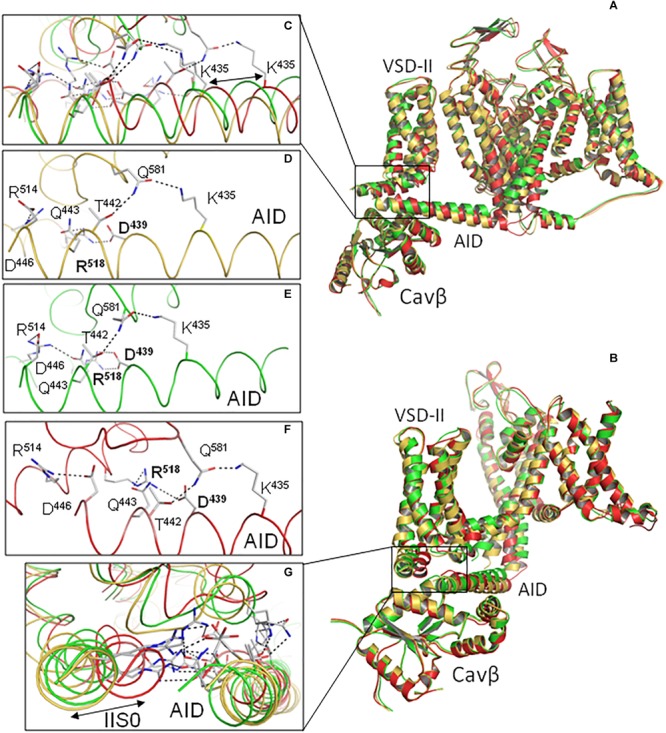
H-bonds and salt bridges between AID and VSD-II are formed in different-state models. Shown are superposed Cav1.1-Ia based models in the inactivated (yellow), open (green), and closed (red) states. **(A)** Side view with VSD-I, IS4-S5 and a part of PD removed for clarity. **(B)** View along AID with repeats III and IV removed for clarity. **(C–F)** Expansions of **(A)** show the network of state-independent H-bonds and salt bridges between AID and VSD-II in the superposed models **(C)** and in models of the inactivated **(D)**, open **(E)**, and closed **(F)** channel. Significant shift of K^435^ between the open and closed states **(C)** illustrates the AID helix shift along its axis. **(G)**, Expansion of **(B)** shows three superposed models. Note a large shift of IIS0 in the direction, which is normal to the IIS0 axis, but small displacement of the AID helix in the direction, which is normal to the AID axis. The network of salt bridges and H-bonds between AID and VSD-II caused the big shift of IIS0 upon the *in silico* PD closure. Upon VDI the same H-bonds would transfer perturbations in deactivating VSD-II to the AID gating-brake motion.

The AID helix is amphiphilic. The helix face, which interacts with VSD-II, contains predominantly acidic, basic, and polar residues (red, blue, and green labels in Figure 6A). Long side chains of these residues form salt bridges and H-bonds with long side chain counterparts in VSD-II. Interestingly, *in silico* closure of the PD caused a significant shift of the AID helix. The shift along the AID helix (see, e.g., positions of K^435^ in Figure 7A,C) is much larger than the shift normal to the helix axis (Figure 7B,G). The AID-IIS0 polar contacts are generally conserved in the inactivated, open, and closed-state modes. For example, the state-independent contacts include salt bridges R^518^ : D^439^, R^514^ : D^444^ and H-bonds K^435^ : Q^581^ and Q^581^ : T^442^ (Figure 7D,E). Due to these contacts, when the gating-brake AID moved upon the *in silico* channel closure, it shifted IIS0 (Figure 7B,G).

**FIGURE 8:**
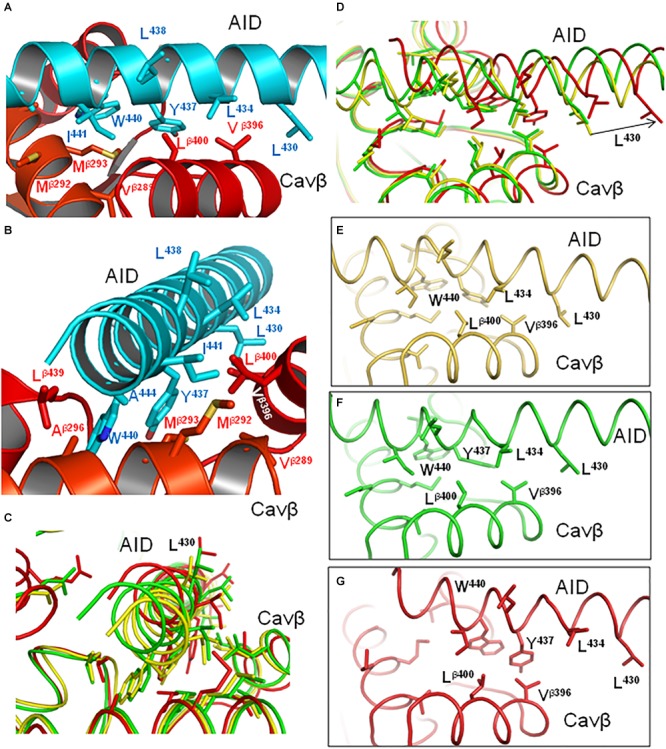
Hydrophobic contacts of AID with Cavβ are state-dependent. **(A,B)** Model ^i^Cav1.2-Ia (cyan AID and red Cavβ) viewed at different angles. The greasy interface between AID and Cavβ would allow the AID gating brake to slide over the Cavβ bench. **(C,D)** Superimposed models ^i^Cav1.2-Ia (yellow), ^o^Cav1.2-Ia (green), and ^c^Cav1.2-Ia (red) viewed at different angles. The arrow in **(D)** shows direction in which L^430^ (as well as entire AID) moves upon *in silico* closure of the channel from the inactivated state. **(E–G)** Individual states from **(D)** show hydrophobic contacts that are modified, switched, or lost upon the *in silico* closure of the channel.

In contrast to the polar face of AID, the AID face, which interacts with Cavβ, contains many hydrophobic or predominately hydrophobic residues (Figure 6B, 8A,B). As already mentioned, the *in silico* PD closure significantly shifted the AID helix along its axis (see relocations of K^435^ in Figure 7C and L^430^ in Figure 8D), but not normally to it (Figure 8C). However, unlike long polar residues in the AID face, which preserved strong contacts with VSD-II (Figure 7C–G), many hydrophobic contacts between AID and Cavβ were modified and some residues switched partners. For example, contacts L^β400^ : W^440^, L^β400^ : Y^437^ and V^β396^ : L^434^ are seen in the open state (Figure 8F), whereas in the closed state Y^437^ switched contact to V^β396^, L^434^ lost contact with Cavβ and L^β400^ slid along W^440^ (Figure 8G). Thus, upon the *in silico* Cav1.2 closure, AID slid over Cavβ and hydrophobic contacts reduced friction, but maintained close contacts of AID with Cavβ.

**FIGURE 9:**
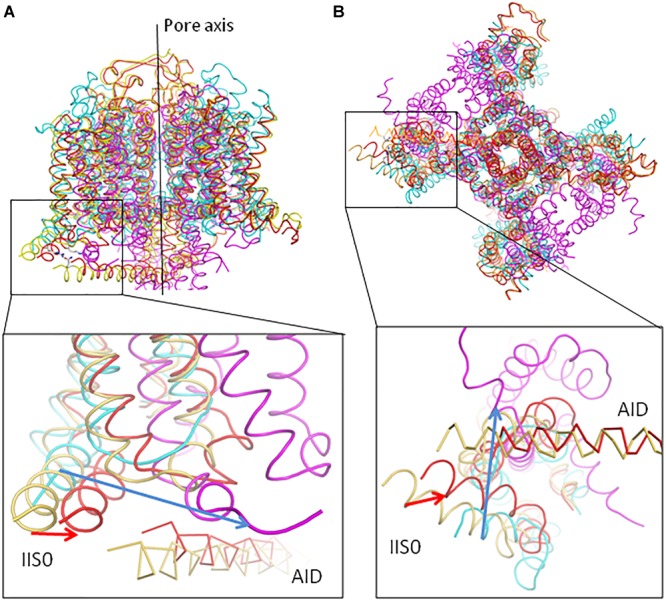
Upon deactivation of VSDs, helices IIS0 in Cav1.2 and S0 in potassium channels shift in comparable directions. Models ^i^Cav1.2-Ia (yellow) and ^c^Cav1.2-Ia (red) are superposed with the X-ray structures of the two-pore potassium channel TPC-1, which has resting-state VSDs (PDB code 5E1J, magenta) and chimeric potassium channel Kv1.2-Kv2.1, which has activated-state VSDs (PDB code 2R9R, cyan). The structures are 3D aligned by minimizing RMS deviations of C^α^ atoms in the P1 helices. The direction of the IIS0 displacement from ^i^Cav1.2-Ia to ^c^Cav1.2-Ia (red arrow) generally concords with that from Kv1.2-Kv2.1 to TPC-1 (blue arrow). In the expanded views, AID in Cav1.2 is shown as C^α^ tracing. **(A)** Side view. **(B)** Cytoplasmic view.

The directions of significant shifts of AID along its axis (Figure 7C, 8D) and IIS0 shift normally to its axis are due to several factors: (*i*) direction of the IS6 C-end shift upon the channel closure (Figure 5F), (ii) strong state-independent contacts between these segments (Figure 6A), and (*iii*) shape peculiarities of the AID, Cavβ and IIS0. Subunit Cavβ has a groove that accommodates AID. The latter can move along the groove but not normally to it. Shift of IIS0 is forced by the strong contacts with AID, but since IIS0 is a part of the big VSD-II, it does not follow direction of the AID shift, but moves at an angle to its axis, causing certain changes in VSD-II. The direction of the IIS0 shift (Figure 9) generally concurs with that in the superposed X-ray structures of two potassium channels: a two-pore potassium channel TPC-1 with VSDs in the resting state (Guo et al., 2016) and chimeric potassium channel Kv1.2/Kv2.1 with VSDs in the activated, “up” state (Long et al., 2007).

### *In silico* Deactivating the Open Channel Without Constraints Between AID and VSD-II

We further employed steered MCM to deactivate model ^o^Cav1.2-Ia by moving alpha carbons in helices S4–S5, S5, and S6 toward matching atoms in ^c^Cav1.2-Ia. In these modeling of the large-scale conformational transition, no constraints between VSD-II and AID were imposed, but maximal separation between the AID and Cavβ backbones was limited to 9 Å. The steered MCM did not destroy contacts between AID and VSD-II; AID moved away the pore axis and shifted VSD-II. The model, which was *in silico* deactivated from open-state model (not shown) was similar to the closed-state model ^c^Cav1.2-Ia. These computations without biasing contacts AID/VSD-II further support important roles of R^518^ and other basic residues in IIS0 in coupling voltage-dependent perturbations in VSD-II with the AID shift.

In contrast to the unbiased modeling of the channel deactivation from the open state, *in silico* closure of inactivated state (see section “Cav1.2 Models in the Open and Closed States”) did require constraints between R^518^ to Q^443^. This is likely because contacts Cavβ/VSD-II and Cavβ/AID in the inactivated-state model, which is based on the cryo-EM structure, are stronger than analogous interdomain contacts in the open-state model, which is based on two different templates, Cav1.1 and NavAb.

### Mutations R518C/H Weaken Contacts AID/VSD-II in the Open- and Closed-State Models

Substitution of the strong H-bond donor R^518^ with histidine or cysteine would obviously affect the IIS0/AID contacts. We are not aware of channelopathies associated with mutations other than R^518^C/H suggesting that such mutations, if happen, are fatal. Mutations R^518^C/H are damaging, but not fatal likely because they weaken contacts of IIS0 with AID and therefore decelerate VDI, but do not eliminate it. To explore such contacts, we modeled mutants R^518^C and R^518^H in the inactivated, open and closed states. Double shell models were used in these computations (see section “Modeling Cav1.2 Mutants”).

**FIGURE 10:**
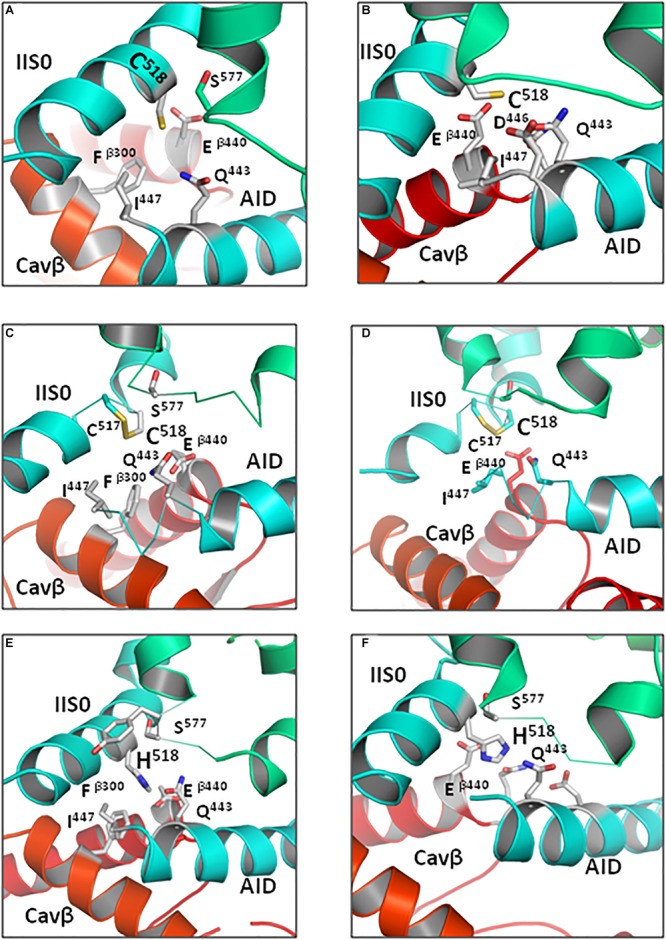
Contacts of R^518^C/H substitutions with AID are weaker than those of wild-type R^518^. Intersegment neighbors within 5 Å from R^518^C/H in the Cav1.2-Ia models are shown by sticks. Models of the open **(A)** and closed **(B)** states with reduced C^518^. Models of the open **(C)** and closed **(D)** states in which oxidized cysteines C^518^ and C^517^ are disulfide-bonded. Open-state **(E)** and closed-state **(F)** models with protonated H^518^. In both open- and closed-state models substitutions of R^518^ interact with Q^443^ and residues at the AID C-end (Tables 2, 3). The weaker contacts would still mediate VDI in the mutants, although less profoundly.

Adjacent cysteines C^517^ and C^518^ may exist in the reduced or oxidized forms. The oxidized cysteines may form a vicinal disulfide bond. Such bonds are found, although infrequently, in proteins (Richardson et al., 2017). Basic residues R^514^ and K^522^, which are located at the same face of the IIS0 helix as C^517^ and C^518^, would repel protons and thus increase probability of the cysteines oxidation. We explored contacts of C^518^ in the models where both adjacent cysteines, C^517^ and C^518^, were either reduced or oxidized. To relax strains in the vicinal disulfide, C^517^ and C^518^ were modeled with flexible bond angles and their alpha carbons were unpinned. In models ^i/o/c^Cav1.2-Ia, both reduced and oxidized cysteine C^518^ accepted an H-bond from Q^443^ in AID (Figure 10A–D and Table 2), suggesting that such contacts only weakly depend on the channel state. H-bonds S—HN are found in small molecules and proteins, see Rossokhin et al. (2011) and references therein. Deprotonated sulfur atoms in disulfides are even more likely to accept H-bonds. For example, in arabinofuranosidase (PDB index 1WD3), the sidechain amide group of N^398^ is at the H-bonding distance from C^401^, which is disulfide-bonded with C^439^. In the same structure, hydroxyl of serine S^299^ is located at the H-bonding distance from cysteine C^176^ that forms a vicinal disulfide with cysteine C^177^. Future analysis of double mutation R^518^C/C^517^A may help exploring a possibility of disulfide bonding between C^517^ and C^518^.

**Table 2:** Energy (kcal/mol)^a^ of intersegment contact involving R^518^C in the inactivated (i), open (o), and closed (c) state models.

Contact^b^		Reduced C^517^ and C^518^						Vicinal disulfide C^517^–C^518^					
		Cav1.2-Ia			Cav1.2-II			Cav1.2-Ia			Cav1.2-II		
		i	o	c	i	o	c	i	o	c	i	o	c
S	577	–0.86	–0.58	–0.80				–0.27	–0.84	–0.70			
L	578					–0.47		–0.13			–0.34	–0.45	
D	439	(–6.5)	(–6.7)	(–3.4)									
Q	443	–0.26	–0.38	–0.62		–0.37		–0.41	–0.87	–0.27		–0.28	–0.3
		(–1.5)	(–1.7)	(–2.6)	(–2.0)		(–3.6)						
D	446			–0.35									
		(–6.1)				(–6.2)	(–5.1)						
I	447		–0.27	–0.61						–0.41			
		(–1.4)		(–0.7)		(–0.6)	(–1.2)						
F	β300	–0.36	–0.26					–0.98	–0.42				
		(–1.1)											
E	β440	–0.50	–1.06	–2.21				–1.67	–1.20	–1.98			
		(–4.2)											

*Interaction energies of wild-type R^*518*^ in models with reduced cysteines (Supplementary Tables S1, S2) are shown in brackets. ^*a*^Interactions with —E— < 0.3 kcal/mol are not shown. ^*b*^Contacts with AID are highlighted.*

Histidine residues exist in protonated and deprotonated forms. All the three tautomeric forms of R^518^H contacted Q^443^ in our models (Table 3). The protonated R^518^H formed contacts with Q^443^, other residues at the C-end of AID, and with Cavβ (Figure 10E,F and Table 3), but these contacts are weaker than those of the WT R^518^ (Supplementary Tables S1, S2). Thus, our calculations predict that, while strong state-independent contacts of R^518^ with AID (Figure 6A) are weakened in substitutions R^518^C/H, the latter form moderate-strength attractive contacts with AID in the inactivated, open and closed-state models. This result agrees with the fact that mutations R^518^C/H have damaging, but not fatal effects on the Cav1.2 physiology. Indeed, mutations, which are associated with channelopathies, are found in patients who suffer, but live. Life is incompatible with non-functional calcium channels.

**Table 3:** Energy (kcal/mol)^a^ of intersegment contact involving R^518^H in the inactivated (i), open (o), and closed (c) Cav1.2-Ia models.

Contact^b^		Tautomer								
		H-N^δ1^			H-N^𝜀2^			H-N^δ1^, H-N^𝜀2^		
		i	o	c	i	o	c	i	o	c
Y	576	–0.45	–0.47						–0.31	
S	577	–2.7	–2.28	–1.20	–2.26	–2.73	–1.45	–3.31	–1.51	–1.37
L	578	–0.62			–0.34			–0.90		
D	339							–0.86	–1.37	–1.46
Q	443	–0.54	–1.18	–1.95	–0.81	–1.52	–1.56	–0.99	–2.65	–1.55
D	446							–0.57	–0.83	–1.24
I	447		–0.64	–1.64		–0.59	0.71		–0.58	–1.16
R	4562		–0.47							0.35
K	2554									0.45
K	β 295							0.40	0.48	
D	B 299							–0.45	–0.44	
F	β300		–0.88		–1.25	–0.61		–0.91	–1.23	
Q	β438			–1.13			–1.61			–1.55
E	β440	–0.72	–1.90	–3.48	–0.88	–2.23	–3.05	–3.94	–6.70	–7.74
D	β441								–0.33	–0.73
E	β444							–0.54		–0.44

*^*a*^Contacts with sequentially adjacent residues and contacts with energy —E— < 0.3 kcal/mol are not shown. ^*b*^Contacts with AID are highlighted.*

### Mutations G^402^S and G^406^R Stabilize the Open-State Model

In the closed-state model ^c^Cav1.2-Ia, G^402/1i24^ contacted large phenylalanine F^1401/4k11^, while G^406^ lacked strong intersegment contacts (not shown). In the open-state model ^o^Cav1.2-Ia, G^402^ and G^406^ formed contacts with a large leucine L^276/1o6^ and small alanine A^272/1o2^, respectively. These results imply that in both open and closed states of the WT channel glycines G^402^ and G^406^ have approximately similar-strength contacts. Thus, likely role of these glycine is not to stabilize or destabilize a specific state of the channel, but to provide flexibility to the cytoplasmic part of helix IS6, which contributes to the activation gate. This flexibility would facilitate the activation gate closure during VDI.

**FIGURE 11:**
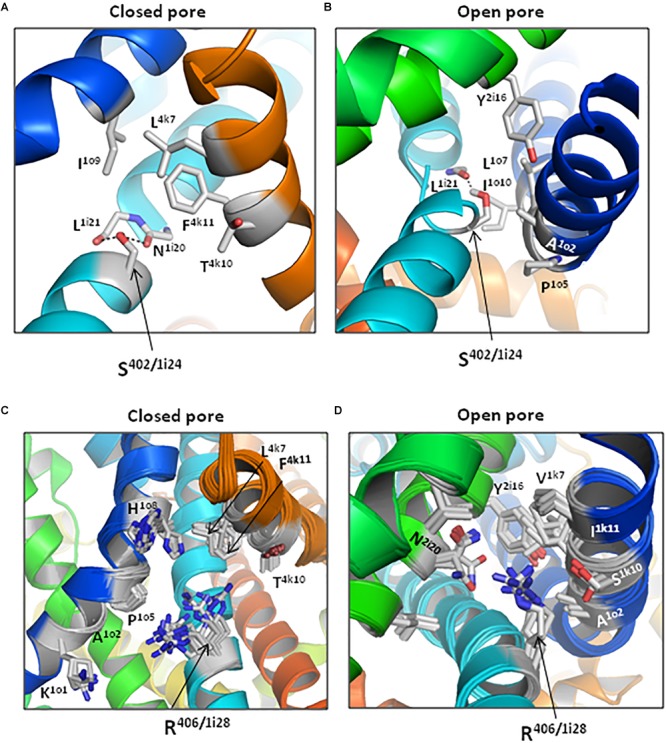
Contacts of substitutions G^402^S and G^406^R are state-dependent. (Universal residue labels are shown in Table 1 and energies of inter-residue contacts are given in Table 4.) Lowest-energy structures with S^402/1i24^ and its neighbors in models ^c^Cav1.2-Ia **(A)** and ^o^Cav1.2-Ia **(B)**. The backbone around S^402^ is not shown for clarity. In the closed channel, S^402^ donates a bifurcate H-bond to the backbone carbonyls of N^1i20^ and L^1i21^. In the open state, S^402^ donates a single H-bond at the kink of IS6. Ensembles of structures within 7 kcal/mol from the apparent global minima in models ^c^Cav1.2-Ia **(C)** and ^o^Cav1.2 **(D)**. Arginine R^406/1i28^ and its neighbors are shown by sticks.

Mutual disposition of the PD helices S4–S5, S5, and S6 in our open- and closed-state models ^o/c^Cav1.2-Ia and ^o/c^Cav1.2-II is inherited from the X-ray structures of open and closed NavAb channel. Therefore, we explored state-dependent intersegment contacts of G^402^S and G^406^R only in models ^o/c^Cav1.2-Ia (Figure 11 and Table 4).

**Table 4:** Energy (kcal/mol) of intersegment contacts of G^402^S and G^406^R in the closed (o) and open (o) models of Cav1.2-Ia^a^.

Contacting	
residue		Label	G^402^S		G^406^R	
			c	o	c	O
I	261	1k11				–2.27
S	260	1k10				–1.38
A	262	1o2		–1.05		–0.63
N	746	2i20				–0.56
V	257	1k7				–0.38
L	269	1o6		–0.82		
P	267	1o5		–0.85	–0.56	
K	261	1o1			0.35	0.33
A	262	1o2			–0.30	
L	1397	4k7			–0.30	
F	1401	4k11	–0.75			
		Σ	–0.75	–2.72	–0.81	–4.98

*^*a*^Energies are given for lowest-energy structures found in MC-minimizations of double-shell models built around the indicated residues. Contacts with interaction energy —E— < 0.3 kcal/mol are not shown.*

Serine S^402/1i24^ forms closed-state non-polar contact with F^4k11^ and open-state non-polar contacts with A^1o2^, P^1o5^ and L^1o6^ (Figure 11A,B and Table 4). Several other residues approach S^402/1i24^ in the closed [I^1o9^, L^4k7^, and T^4k10^ (Figure 11A)] or open [L^1o7^, I^1o10^, and Y^2i16^ (Figure 11B)] states, but respective interaction energies are weak and they are not shown in Table 4. In the closed channel, the side chain of S^402/1i24^ donates H-bonds to the backbone carbonyls of N^1i20^ and L^1i21^, but due to tight packing of the S6 bundle in these H-bonds do cause a noticeable bend in IS6 (Figure 11A). In the open state, IS6 is bent (Figure 11B) and the H-bond between the S^402/1i24^ sidechain and the backbone carbonyl of L^1i21^ may contribute to the bending. The net energy of moderate-strength intersegment contacts of S^402/1i24^ is -0.75 kcal/mol in the closed state and -2.72 kcal/mol in the open state (Table 4).

In the closed-state model ^c^Cav1.2-Ia, arginine R^406/1i24^ forms attractive non-polar contacts with P^1o5^, A^1o1^, and L^4k7^. Besides, several other residues are within 5 Å from R^406^ (Figure 11C), but energies of these contacts are small. In the open channel, R^406^ forms van der Waals contacts with three residues (I^1k11^, A^1o2^ and V^1k7^) and polar contacts with N^2i20^ and S^1k10^ (Figure 11D). In both open and closed states, R^406^ experiences electrostatic repulsion from K^1o1^. The net energy of moderate and strong intersegment contacts of R^406/1i24^ is -0.81 kcal/mol in the closed state and -4.98 kcal/mol in the open state (Table 4).

Thus, in the closed channel both G^402^S and G^406^R have fewer contacts than in the open channel (Figure 11) and the closed-state contacts are significantly weaker than the open-state contacts (bottom line in Table 4). The stronger open-state contacts of the mutants would resist and therefore slow-down the pore closure upon VDI in agreement with experimental data (Barrett and Tsien, 2008).

## Discussion

### Cardiology

Genetic variants of gene *CACNA1C* are associated with a variety of clinical conditions mostly linked to abnormal calcium cycling, cell excitation, and propagation of action potential. The gene is widely expressed in many tissues including brain, kidney, and endocrine glands with highest expression level in cardiac and smooth muscles. As a consequence, most of *CACNA1C* mutations are associated with cardiac arrhythmic and structural disorders such as long QT syndrome, Brugada syndrome, paroxysmal familial ventricular fibrillation or hypertrophic cardiomyopathy. Of note, such a wide spectrum of cardiac functional and structural diseases is rarely seen in association to one single gene with an exception of *SCN5A*-encoded cardiac Nav1.5 channel, a homolog of Cav1.2. The widest spectrum of tissues and organs involved in Cav1.2-associated conditions is observed in TS, a rare disorder which includes alterations in central nervous system, heart and limb development as well as immune system defects. The fact that this multisystem condition is almost exclusively associated with mutations G^406^R and G^402^S reflects the functional importance of these amino acid residues for channel function in most of cell and organs. Additional R^518^C/H mutations described in our and several other studies in association with cardiac-only TS underline the complexity of functional effects due to this amino acid substitutions as well. Taking into account the high risk of sudden cardiac death due to Cav1.2 dysfunction in all above mentioned clinical conditions, the detailed studies of structural channel rearrangements due to these mutations are important for cardiology and general physiology.

### Cav1.2 Models With the Open and Closed Pore

The ion-permeation pathway in the cryo-EM structure Cav1.1 is closed (Wu et al., 2016). To model Cav1.2 with the open and closed PD we used as templates the X-ray structures of a prokaryotic sodium channel NavAb with the open (PDB code 5vb8) and closed (PDB code 5vb2) activation gate (Lenaeus et al., 2017). Employment of a symmetric homotetrameric channel to model a pseudo-heteromeric eukaryotic sodium channel is questionable, but other templates seem even less appropriate for modeling large-scale conformational transitions of PD. Several arguments support the choice of NavAb. (i) Eukaryotic calcium channels are more related to homotetrameric prokaryotic sodium channels than to homotetrameric potassium channels. (ii) Open- and closed-state cryo-EM structures of another prokaryotic sodium channel, NavCt (Tsai et al., 2013) are of low resolution and they lack linker helices S4–S5. (iii) In the cryo-EM structures of eukaryotic sodium channels NavPaS (Shen et al., 2017), EeNav1.4 (Yan et al., 2017), and hNav1.4 (Pan et al., 2018), the PD is asymmetric and the activation gate is not wide enough to let through large bulky ligands like batrachotoxin, which targets the inner pore of both eukaryotic (Tikhonov and Zhorov, 2005; Du et al., 2011) and prokaryotic (Finol-Urdaneta et al., 2018) sodium channels. (iv) Mutual disposition of cytoplasmic halves of S6 helices, which form the activating gate, is much more similar between channel Cav1.1, NavPaS, and *Ee*Nav1.4 (Supplementary Figure S5A) than between channels Cav1.1 and NavAb, especially in the closed state of the latter (Supplementary Figure S5B). Therefore, the available structures of eukaryotic sodium channels are inappropriate for modeling the closed-state Cav1.2. (v) Despite the fact that closed-state structure of NavAb is stabilized by introducing two mutations in segments S6 (Lenaeus et al., 2017), the pore dimensions of the closed NavAb are similar to those of the closed prokaryotic potassium channel KcsA (Doyle et al., 1998) (Supplementary Figure S6).

In the superposed models ^o^Cav1.2-Ia and ^c^Cav1.2-Ia the backbone shift of Cavβ is small (Figure 4A). Various data suggest that the *in vivo* mobility of Cavβ is limited. (i) The membrane anchorage of Cavβ is critical for the channel function (Restituito et al., 2000; Suh et al., 2012). (ii) The Cav1.2 channels coexpressed with not palmitoylated, but membrane-attached Cavβ2e have properties, which are similar to the channels coexpressed with Cavβ2a (Takahashi et al., 2003). (iii) Ablation of a Cav1.2-anchoring protein, which interacts with the cytoplasmic face of the channel, restores the normal Cav1.2 function in TS-associated mutants (Cheng et al., 2011). These data suggests that TS develops in the channels with restrained mutual disposition of the α1 and β subunits.

### Structural Mechanism of VDI

The key role of the I/II linker in the calcium channel inactivation has long been recognized (Bourinet et al., 1999; Stotz and Zamponi, 2001). In lack of high-resolution structures, the N-end part of the linker was suggested to plug the pore by analogy with the hypothetical mechanism of hinged-lid block of sodium channels during fast inactivation (Stotz et al., 2004). In the cryo-EM structures of Nav1.4 (Shen et al., 2017; Pan et al., 2018), the fast-inactivation IFM motif does not plug the pore, but binds in the interface between helices IIIS4-S5, IVS4-S5, IIIS6, and IVS6 (Supplementary Figure S7A). In both the closed- and open-state models of Cav1.2, linker I/II does not occlude the pore (Supplementary Figures S7B,C). Rather, it is displacement of AID toward the pore axis that would shift the C-end of AID-linked IS6, initiating the activation gate closure. Thus, the analogy between fast inactivation in sodium channels and VDI in Cav1.2 still exists. In both cases the linkers not form “hinged lids,” but instead they mediate shifts of cytoplasmic parts of S6 helices toward the pore axis, thus allosterically initiating the activation gate closure.

Arginine R^518^ and other basic residues in helix IIS0 form strong contacts with AID in all our models (Figure 3, 6 and Supplementary Tables S1, S2), implying that R^518^ plays an important, although not a unique role in maintaining contacts between VSD-II and AID in different states. These contacts are apparently of key importance during VDI when IIS0 pushes the AID “gating brake” toward the pore axis, initiating the activation gate closure.

Based on our models we suggest the following six-step mechanism of VDI. (i) Upon membrane depolarizations S4 helices shift in the extracellular direction and the activation gate opens up. (ii) The shift of IIS4 causes perturbations within VSD-II that involve helix IIS0 and linker IIS2-S3 (Figure 9). (iii) Due to strong contacts of IIS0 and IIS2-S3 with AID, the latter moves toward the pore axis. (iv) When AID moves, its polar contacts with IIS0 are maintained, whereas hydrophobic contacts with Cavβ are switched to minimize friction. (v) The AID-linked helix IS6 transits from the open- to closed-state conformation and flexible G^402^ and G^406^ facilitate the transition. (vi) The IS6 transition initiates cooperative transitions of other transmembrane helices in PD to the closed-state conformations.

The closed-state Cav1.2 models, which are obtained by targeting the PD helices toward positions in the symmetric NavAb, are substantially different from our inactivated-state models, which are based on the asymmetric cryo-EM structures of Cav1.1. The closed-state models do not represent the resting state in which helices S4 should be in the “down” position. Besides the resting, open, and inactivated states, voltage-gated ion channels adopt various intermediate states whose structures are unclear. We suggest that the activation gate closure is a necessary step in developing VDI.

The proposed mechanism is consistent with and integrates multiple experimental observations. A key role of VSD-II in the channel activation is consistent with the data that VSD-II and VSD-III provide ∼85% of the total energy toward stabilizing the Cav1.2 open state (Pantazis et al., 2014). VDI causes the gating charge immobilization (Barrett and Tsien, 2008). Mutations G^402^S and G^406^R, which in our models stabilize the open-state conformation of IS6, delay fast development of VDI (Barrett and Tsien, 2008). Mutations R^518^C/H, which in our models weaken contacts of IIS0 with AID, cause the loss of current density, increase window and late current, and decelerate VDI (Boczek et al., 2015b). On the other hand, mutations R^518^H/C cause only a small shift in the activation curves (Boczek et al., 2015b) consistent with our models where these mutations do not affect relative stability of the open and closed states. Close contacts of R^518^ with D^439^ and Q^443^ (Figure 6A, 7D–F) are consistent with the fact that mutations D^439^R, T^442^D, and Q^443^K/R (respective residues in rabbit Cav1.2 are 469, 472, and 473) exhibit the largest fraction of whole-cell currents at the end of a conditioning depolarization pulse and these currents are two to three times larger than those in WT channels, see Table 1 in Dafi et al. (2004). Strong AID-Cavβ contacts in our models (Figure 6B, 8) are consistent with the data that mutations of Y^437^, W^440^, and I^441^ greatly reduce Cavβ/AID interactions and Cavβ-induced stimulation of calcium currents (Buraei and Yang, 2013).

Residues R^514^ and R^518^ in helix IIS0 make the strongest contacts with AID (Figure 6A and Supplementary Tables S1, S2). We are not aware of Cav1.2 dysfunctions associated with mutations of R^514^. Arginine R^514^ is farther than R^518^ from the turn between helices IIS0 and IIS1. Therefore, upon perturbation of VSD-II, contacts of R^518^ are likely to shift AID, while contacts of R^514^ at the N-end of IIS0 would produce a large momentum, which would cause deformations of IIS0. Such deformations are seen in Class II cryo-EM structure of Cav1.1 (Wu et al., 2016).

Our Cav1.1-Ia based structures form stronger AID/VSD-II contacts, but weaker AID/Cavβ contacts (Supplementary Table S1). In lack of experimental structures with the full-fledged linker I/II and full-fledged Cavβ, interpretation of these data would be premature.

### Cavβ and VDI

Tight contacts between Cavβ and AID have long been known (see Van Petegem et al., 2004) and references therein. Various studies indicate that these interactions play important role in the channel modulation (Minor and Findeisen, 2010). *In vitro*, Cavβ2a causes a hyperpolarizing shift of VDI (∼10–20 mV) and inhibits VDI in Cav1.2 (Buraei and Yang, 2010). However, in other experiments presence or absence of Cavβ did not affect the VDI kinetics (Barrett and Tsien, 2008).

Mutations G^402^S and G^406^R retard inactivation of Cav1.2 coexpressed with either β2a or β1c subunit, but inactivation with β1c is much faster than that with β2a (Barrett and Tsien, 2008). In model ^i^Cav1.2-Ia, R^515^ forms a salt bridge with D^β306^ (Supplementary Table S1). In homologous positions, β1c and β2a have D^β261^ and E^β259^, respectively (Supplementary Figure S2). Contacts of R^515^ with the acidic residues may contribute to different effects of β1c and β2a on the Cav1.2 inactivation kinetic.

Strong contacts between VSD-II and Cavβ (Supplementary Table S1) would resist perturbations at the cytoplasmic face of VSD-II and thus displacement of VDI over Cavβ. Such contacts are stronger in ^i^Cav1.2-Ia than in ^i^Cav1.2-II, whereas contacts of Cavβ with AID are stronger in ^i^Cav1.2-II than in ^i^Cav1.2-Ia. Contacts of mobile VSD-II and AID with a less mobile Cavβ would resist VDI. Essentially different energies of the Cavβ contacts in our inactivated-state models model suggest that respective cryo-EM structures captured the Cav1.1 channel in two different sub-states that may be populated in the process of VDI.

### Effects of Mutations on Cav1.2 Functions

Mutation A^582^D in the IIS2-S3 cytoplasmic loop of VSD-II causes a gain-of-function syndrome LQTS-8 (Fukuyama et al., 2014). Alanine A^582^ forms contacts with T^442^ in model ^o^Cav1.2-II and with D^439^ in model ^o^Cav1.2-Ia (Supplementary Table S2). Mutation A^582^D would fortify contacts with the polar residues, thus stabilizing the open state. Mutation D^446^G in AID causes a LQTS listed in ClinVar (Landrum et al., 2018). In both open- and closed-state models, ^o^Cav1.2-Ia and ^c^Cav1.2-Ia, aspartate D^446^ forms strong contacts with R^514^ (Figure 6A). Elimination of this contact would have effects, which are similar (but not identical) to the effects caused by mutations R^518^C/H.

Conserved mutations D^448^N and E^450^Q in positions immediately C-terminal to the resolved part of AID, cause LQTS syndromes described in ClinVar. Our models suggest that these acidic residues may form salt bridges with R^511^, which is located at the N-end of IIS0. Interestingly, mutation R^511^Q, which would destroy the salt bridges, is also listed in ClinVar, although its clinical consequences are unclear. These data support our conclusion that arginines in IIS0 stabilize interaction of VSD-II with AID and Cavβ.

Mutations G^402^A/M/N/V/W in the rabbit Cav1.2 channel reduced the channel inactivation (Depil et al., 2011). A KcsA-based homology model of the closed-state Cav1.2 channel further predicted that G^402^ is involved in tight S6–S6 packing, suggesting that substitutions of G^402^ with larger residues would destabilize the closed state (Depil et al., 2011). In our model of the closed Cav1.2, G^402^ does not form tight contacts with neighboring S6 helices (Table 4) implying that it is flexibility of IS6, which is important for the fast VDI. Substitutions of G^402^ with larger residues would reduce this flexibility.

A model of Cav1.2 channel with mutation Lys773del, which is associated with a calcium channelopathy (Rafiq et al., 2017), shows significant structural alterations vs. WT channel that are hardly consistent with a functional channel. Lysine K^773^ is located before cytoplasmic linker II/III (Supplementary Figure S1), which is not resolved in the Cav1.1 cryo-EM structures. Deletion of K^773^ likely shifts the conformational equilibrium of the II/III linker resulting in the channel proteins, which are malfunctioning, but still compatible with life.

Besides the TS-associated mutations, which are considered in this study, the ClinVar database (Landrum et al., 2018) describes many Cav1.2 mutations, which are associated with various channelopathies. Some of these mutations are studied electrophysiologically (e.g., Splawski et al., 2004, 2005; Antzelevitch et al., 2007; Fukuyama et al., 2014; Wemhoner et al., 2015). Further studies are necessary to explore possible structural consequences of these and other well-characterized mutations in Cav1.2.

### Limitations of the Modeling Approach

In this study we have built models of the human Cav1.2 channel in the inactivated, open and closed states. These are based, respectively, on cryo-EM structures of the presumably inactivated rabbit Cav1.1 channel (Wu et al., 2016) and X-ray structures of prokaryotic sodium channel NavAb in the open and closed states (Lenaeus et al., 2017). Since the experimental structures are obtained in lack of lipid membranes and membrane voltage, some of their features may be non-native. In addition, because the open- and closed-state conformations of the Cav1.2 channel combine structural features from both Cav1.1 (VSDs and the extracellular half of PD) and NavAb (the intracellular half of PD), the homology modeling could have increased the likelihood of non-native conformations. Furthermore, in lack of experimental structural data on the C-terminal part of linker I/II we refrained from an attempt to *de novo* model this part, which appears to be important for function. Despite these limitations, which are mainly due to insufficient structural data, our models are consistent with a large number of experimental observations, which are described the Discussion.

## Conclusion

Here we reported a case of cardiac-only TS caused by mutation R^518^C and aimed to explore possible atomic-level mechanisms of this and other TS-associated mutations. Toward this goal we compared open- and closed-state models of the Cav1.2 channel and analyzed contacts of residues R^518^, G^402^, G^406^ and their TS-associated substitutions R^518^H/C, G^402^S, and G^406^R. Arginine R^518^ and several other residues at the cytoplasmic face of VSD-II form strong contacts with the AID in all our models. We propose that, following the voltage-dependent channel activation, the cytoplasmic face of VSD-II would perturb and shift IIS0-bound AID toward the pore axis. The AID-linked IS6 would bend at flexible G^402^ and G^406^, facilitating the activation-gate closure and thus VDI. Mutations R^518^C/H would weaken the IIS0-AID contacts, retarding the AID displacement. Mutations G^406^R and G^402^S would stabilize the open state, thus resisting the pore closure upon the AID shift. Taken together, our results provide a mechanistic rationale for the VDI deceleration by TS-associated mutations and suggest targets for mutational and electrophysiological studies of calcium channelopathies and future gene editing.

## Author Contributions

AAK and BZ initiated the research. AAK and EM performed medical and electrocardiographic examination. AMK performed genetic studies and validation. BZ and VK performed computations. BZ wrote the manuscript with contributions from VK and AAK.

## Conflict of Interest Statement

The authors declare that the research was conducted in the absence of any commercial or financial relationships that could be construed as a potential conflict of interest.

## Abbreviations

AID: α1-interaction domain
Cav1.1-Ia and Cav1.1-II: Class Ia and Class II cryo-EM structures of Cav1.1
^i/o/c^Cav1.2-Ia and ^i/o/c^Cav1.2-II: inactivated-, open- or closed- state models based, respectively, on Cav1.1-Ia, Cav1.1-II, and X-ray structures of the open or closed sodium channel NavAb
Cavβ: calcium channel β-subunit
CDI: calcium-dependent inactivation
CTD: C-terminal domain
p-CTD: proximal CTD
d-CTD: distal CTD
LQTS: long QT syndrome
MC: Monte Carlo
MCM: MC-minimization
PD: pore domain
RMSD: root mean square deviation
TS: Timothy syndrome
VDI: voltage-dependent inactivation
VSD: voltage-sensing domain
WT: wild type
