# Supplementary material for: Atomic Mechanisms of Timothy Syndrome-Associated Mutations in Calcium Channel Cav1.2
Source: Front Physiol. 2019 Mar 29;10:335. doi: 10.3389/fphys.2019.00335 (PMC6449482; doi:10.3389/fphys.2019.00335)
Supplement: Supplementary file 1 [file Data_Sheet_1.PDF]

Table S1. Energy (kcal/mol) of interdomain contacts in inactivated-state models ^{i}Cav1.2-Ia and ^{i}Cav1.2-II. Contacts with energy |E| < 0.4 kcal/mol are not shown.

**Residues**		**Model**	
**AID**	**VSD-II**	**Ia**	**II**
R 435	Q 581	-1.7	
	V 585	-1	-1.1
L 438	Q 581	-0.9	
**D 439**	**R 518**	**-6.5**	
	A 582	-0.9	-0.4
	V 585		-0.9
T 442	Q 581	-0.7	
**Q 443**	**R 518**	**-1.5**	**-2**
	R 514	-1.9	-0.8
D 446	R 514	-6.1	-4.5
I 447	R 514	-1.4	-1.3
	R 514	-1.2	
	**Σ=**	-23.8	-11

**VSD-II**	**Cavβ**	**Ia**	**II**
R 514	D β299	-5.4	
	H β303	-3.1	
	K β302	1.8	
R 515	D β306	-5.4	
	H β303	-3.4	
	D β299		-8.3
**R 518**	**E β440**	**-4.2**	
	**F β300**	**-1.1**	
K 522	E β440	-5.7	-5.1
	E β444	-6	
	D β441	-2.8	
	**Σ=**	-35.3	-13.4

**Residues**		**Model**	
**AID**	**Cavβ**	**Ia**	**II**
L 430	S β393	-1	
	K β395	-0.7	-0.8
	V β396		-0.7
	R β399		-1.6
E 432	N β437	-0.8	-2.4
D 433	N β437	-3.5	-1.8
	V β396	-0.7	-0.9
L 434	L β400	-1	
	R β399	-1.2	-1
Y 437	I β391	-1.8	-1.4
	M β293	-0.9	-0.7
	V β396	-0.7	-0.5
	L β400	-1.1	-1.8
D 439	Q β438	-0.5	-0.9
W 440	A β442	-0.8	-0.6
	A β296	-1.1	-0.7
	L β297	-1.2	-1.1
	I β389	-1.2	
	Q β438	-1.8	
	N β437		-1
	M β293		-4.3
	L β439		-3
I 441	M β293	-0.9	
	M β292	-1	
	R β404		-1
Q 443	L β439	-0.6	-0.4
E 445	M β292	-1.6	-1.8
	R β404		-7.8
I 447	D β299		-1.3
	**Σ=**	-24.1	-37.5

Table S2. Energy (kcal/mol) of AID contacts with VSD-II and Cavβ in the open (Eo) and closed (Ec) models. Contacts with energy |E| < 0.5 kcal/mol are not shown.

Residues			Model			
AID	VSD-II	Cavβ	Ia		II	
			Eo	Ec	Eo	Ec
L 430		R β399	-1.3		-0.9	
		V β396	-0.7			
		K β395	-0.8			
E 431	R 649					-4.4
E 432	V 585		-0.6			
D 433		K β395				-5.2
		V β396	-0.6		-0.5	
		N β437	-1.5		-0.5	
L 434		R β399	-1.4	-1.2		
		V β396	-1			
R 435	F 584		-1.5			
	V 585		-0.8	-0.6		
	Q 581		-4.2	-1.8		
	R 649					1.8
G 436		N β437		-0.7		
Y 437		I β391	-2.1	-0.9	-0.5	
		K β395				-1.1
		V β396	-0.9	-1.9	-0.7	-1.1
		R β399				-2.1
		L β400	-1.2	-0.5	-1.7	
		N β437	-1.4			
		M β293	-1.1			
		T β392		-1.2		
		S β393		-2.1		
		N β437		-0.7		
		Q β438		-0.6		
L 438	Q 581		-1.2	-1.7		
D 439	**R 518**		**-6.7**	**-3.4**		
	Q 581		-0.8	-1.5		
	A 582		-0.9			
		Σ =	-30.7	-18.8	-4.8	-12.1

Residues			Model			
AID	VSD-II	Cavβ	Ia		II	
			Eo	Ec	Eo	Ec
W 440		M β293	-4.6		-0.8	
		I β389	-1.5			
		L β434	-0.5			
		N β437	-0.8	-4.9	-0.9	-1.4
		Q β438	-0.6			
		L β439	-3.2		-1	-0.9
		A β296	-1.2			
		L β297	-0.7			
		I β391		-1.8		
I 441		V β289	-0.5			
		M β292	-1.3			
		M β293	-0.8			
		L β400		-0.7		
T 442	Q 581		-0.7	-1.5		
	A 582				-0.9	
	V 585				-0.7	
Q 443	R 514		-2.8			
	**R 518**		**-1.7**	**-2.6**		**-3.6**
	L 578		-0.9			
		Q β438		-0.5		
A 444		M β292	-0.7			
		K β295	-3.3			
E 445		M β292	-0.6			
D 446	R 514		-6.3	-6.6	-6.5	
	**R 518**				**-6.2**	**-5.1**
	L 578			-2.3		
I 447	R 514		-2.1			
	R 511			-1.4		
	**R 518**			**-0.7**	**-0.6**	**-1.2**
	R 515				-1.5	
		D β299	-1.1		-0.8	
		F β300	-0.5			
		H β303	-0.8			
		Σ =	-37.2	-23	-19.9	-12.2
