# Supplementary material for: Atomic Mechanisms of Timothy Syndrome-Associated Mutations in Calcium Channel Cav1.2
Source: Front Physiol. 2019 Mar 29;10:335. doi: 10.3389/fphys.2019.00335 (PMC6449482; doi:10.3389/fphys.2019.00335)
Supplement: Supplementary file 2 [file Data_Sheet_2.PDF]

Figure S1

```
Cav1.1          ------------------------------------------------------------    0
Cav1.2          MVNENTRMYIPEENHQGSNYGSPRPAHANMNANAAAGLAPEHIPTPGAALSWQAAIDAAR    60
                                                           ~~~~~~~~===1s0===
Cav1.1          -------------MEPSSPQDEGLRKKQPKKPLPEVLPRPPRALFCLTLQNPLRKACISI    47
Cav1.2          QAKLMGSAGNATISTVSSTQRKRQQYGKPKKQGSTTATRPPRALLCLTLKNPIRRACISI    120
                                ** * :  :  :***    .  ******:****:**:*:*****

                ===~~======1s1========~~~~~~~~~~~~=======1s2================
Cav1.1          VEWKPFETIILLTIFANCVALAVYLPMPEDDNNSLNLGLEKLEYFFLTVFSIEAAMKIIA    107
Cav1.2          VEWKPFEIIILLTIFANCVALAIYIPFPEDDSNATNSNLERVEYLFLIIFTVEAFLKVIA    180
                ******* **************:*:*:****.*: * .**::**:** :*::** :*:**

                =~~~~~~~~~========1s3==========~~~~~~~~~~~~~~~~~~~~~~=======
Cav1.1          YGFLFHQDAYLRSGWNVLDFIIVFLGVFTAILEQVNVIQSNTAPMSSKGAGLDVKALRAF    167
Cav1.2          YGLLFHPNAYLRNGWNLLDFIIVVVGLFSAILEQATKAD.GANALGGKGAGFDVKALRAF    239
                **:*** :****.***:******.:*:*:*****..  :  .:  :..****:********

                ==1s4======~~~~~=1L45====~~~~~~~======1s5========~~~~~~~~~~~
Cav1.1          RVLRPLRLVSGVPSLQVVLNSIFKAMLPLFHIALLVLFMVIIYAIIGLELFKGKMHKTCY    227
Cav1.2          RVLRPLRLVSGVPSLQVVLNSIIKAMVPLLHIALLVLFVIIIYAIIGLELFMGKMHKTCY    299
                **********************:***:**:********::*********** ********

                ~~~~~~~~~~~~~~~~~~~~~~~~~~~~~~~~~~~~~~~~~~~~~~~~~~~==1p1====
Cav1.1          YIGTDIVATVENEKPSPCART-GSGRPCTINGSECRGGWPGPNHGITHFDNFGFSMLTVY    286
Cav1.2          NQEG.IADVPAEDDPSPCALETGHGRQC-QNGTVCKPGWDGPKHGITNFDNFAFAMLTVF    357
                     *. .  ::.*****   * ** *  **: *: ** **:****:****.*:****:

                ====~~~===1p2======~~~~~=========1s6====================~~~~
Cav1.1          QCITMEGWTDVLYWVNDAIGNEWPWIYFVTLILLGSFFILNLVLGVLSGEFTKEREKAKS    346
Cav1.2          QCITMEGWTDVLYWVNDAVGRDWPWIYFVTLIIIGSFFVLNLVLGVLSGEFSKEREKAKA    417
                ******************:*.:**********::****:************:*******:

                ~~~======== AID =============~
Cav1.1          RGTFQKLREKQQLEEDLRGYMSWITQGEVMDVEDLREGKLSLEEG-----GSDTESL---    398
Cav1.2          RGDFQKLREKQQLEEDLKGYLDWITQAEDIDPENEDEGMDEEKPRNMSMPTSETESVNTE    477
                ** **************:**:.****.* :* *:  **  . :        *:***:

                                                 ~~~~~~2S0 === =====2S1=====
Cav1.1          ----YEIEG---------LNKIIQFIRHWRQWNRVFRWKCHDLVKSRVFYWLVILIVALN    445
Cav1.2          NVAGGDIEGENCGARLAHRISKSKFSRYWRRWNRFCRRKCRAAVKSNVFYWLVIFLVFLN    537
                     :***           .  :* *:**:***. * **:  ***.*******::* **

                ====~~~~~~~~~~=========2S2============~~~~~~~~~~~======2S3==
Cav1.1          TLSIASEHHNQPLWLTHLQDIANRVLLSLFTIEMLLKMYGLGLRQYFMSIFNRFDCFVVC    505
Cav1.2          TLTIASEHYNQPNWLTEVQDTANKALLALFTAEMLLKMYSLGLQAYFVSLFNRFDCFVVC    597
                **:*****:*** ***.:** **:.**:*** *******.***: **:*:**********

                ===========~~~~=====2s4============~===2L45=======~====2s5==
Cav1.1          SGILELLLVESGAMTPLGISVLRCIRLLRLFKITKYWTSLSNLVASLLNSIRSIASLLLL    565
Cav1.2          GGILETILVETKIMSPLGISVLRCVRLLRIFKITRYWNSLSNLVASLLNSVRSIASLLLL    657
                .**** :***:  *:*********:****:****:**.************:*********

                ================~~~~~~~~~~~~~~~~~~~======2p1====~~~==2p2====
Cav1.1          LFLFIIIFALLGMQLFGGRYDFEDTEVRRSNFDNFPQALISVFQVLTGEDWNSVMYNGIM    625
Cav1.2          LFLFIIIFSLLGMQLFGGKFNFDEMQTRRSTFDNFPQSLLTVFQILTGEDWNSVMYDGIM    717
                ********:*********:::*:: :.***.******:*::***:***********:***
```

```
                    ==~~~~~~~~=========2s6======================================
Cav1.1              AYGGPSYPGVLVCIYFIILFVCGNYILLNVFLAIAVDNLAEAESLTSAQKAKAEERKRRK    685
Cav1.2              AYGGPSFPGMLVCIYFIILFICGNYILLNVFLAIAVDNLADAESLTSAQKEEEEEEKERKK   777
                    ******:**:**********:*****************:********* : **::*:*

Cav1.1              MSRGLPDKTEEEK---SVMAK------KLEQKPKGEGIPTTAKLKVDEFESNVNEVKDPY    736
Cav1.2              LARTASPEKKQELVEKPAVGESKEEKIELKSITADGESPPATKINMDDLQPNENEDKSPY    837
                    ::*    :.::*     .:.:      :*:.   .   * ::*:::*::: * ** *.**

                                                                   ===3s0===
Cav1.1              PSADFPGDDEEDEPEIPVSPRPRPLAELQLKEKAVPIPEASSFFIFSPTNKVRVLCHRIV    796
Cav1.2              PNPETTGEEDEEEPEMPVGPRPRPLSELHLKEKAVPMPEASAFFIFSSNNRFRLQCHRIV    897
                    *. :  *:::*:***:**.******:**:*******:****:*****  .*:.*: *****

                    =~~~~~======3s1=====~~~~~~~~~~~~=====3s2===============...
Cav1.1              NATWFTNFILLFILLSSAALAAEDPIRAESVRNQILGYFDIAFTSVFTVEIVLKMTTYGA    841
Cav1.2              NDTIFTNLILFFILLSSISLAAEDPVQHTSFRNHILFYFDIVFTTIFTIEIALKILGNAD    957
                    * * ***:**:****** :******::  *.**:**   *  **::**:** **

                                        ===========3S3=============       =====
Cav1.1              --------------------FLHKGSFCRNYFNILDLLVVAVSLISMGLESSTISVVKIL    896
Cav1.2              YVFTSIFTLEIILKMTAYGAFLHKGSFCRNYFNILDLLVVSVSLISFGIQSSAINVVKIL    1017
                                        ******************:*****:*::**:*.*****

                    ===3S4======   ===========3s5=========================~~~~~~
Cav1.1              RVLRVLRPLRAINRAKGLKHVVQCVFVAIRTIGNIVLVTTLLQFMFACIGVQLFKGKFFS    956
Cav1.2              RVLRVLRPLRAINRAKGLKHVVQCVFVAIRTIGNIVIVTTLLQFMFACIGVQLFKGKLYT    1077
                    ************************************:******************:::

                    ~~~~~~~~~~~~~~~~~~~~~~~~~~~~~~~~~~~~~~~~~~~~===3P1=====~~~~~=
Cav1.1              CNDLSKMTEEECRGYYYVYKDGDPTQMELRPRQWIHNDFHFDNVLSAMMSLFTVSTFEGW    1016
Cav1.2              CSDSSKQTEAECKGNYITYKDGEVDHPIIQPRSWENSKFDFDNVLAAMMALFTVSTFEGW    1137
                    *.* ** ** **:* * .****:  :  ::**.* :..*.*****:***:*********

                    ==3P2====~~~~~~~~~~~~=========3s6=======================~~~
Cav1.1              PQLLYRAIDSNEEDMGPVYNNRVEMAIFFIIYIILIAFFMMNIFVGFVIVTFQEQGETEY    1076
Cav1.2              PELLYRSIDSHTEDKGPIYNYRVEISIFFIIYIIIIAFFMMNIFVGFVIVTFQEQGEQEY    1197
                    *:****:***: ** **:** ***::********:******************** **

                    ~~~~~~~==============~~~~~~~~~~~~~=====4s0====~~=====4s1=======~
Cav1.1              KNCELDKNQRQCVQYALKARPLRCYIPKNPYQYQVWYVVTSSYFEYLMFALIMLNTICLG    1136
Cav1.2              KNCELDKNQRQCVEYALKARPLRRYIPKNQHQYKVWYVVNSTYFEYLMFVLILLNTICLA    1257
                    ************:********* ***** :**:*****.*:*******.**:******.

                    ~~~~~=======4s2================~~~~~~~~~~~=====4s3========
Cav1.1              MQHYHQSEEMNHISDILNVAFTIIFTLEMILKLLAFKARGYFGDPWNVFDFLIVIGSIID    1196
Cav1.2              MQHYGQSCLFKIAMNILNMLFTGLFTVEMILKLIAFKPKGYFSDPWNVFDFLIVIGSIID    1317
                    **** **  ::   :***: ** :**:******:***  :***.***************

                    =========                                    ===4s4==
Cav1.1              VILSEIDTFLASSGGLYC----LG----------------GGCGNVDPDESARISSAFFR    1236
Cav1.2              VILSETNHYFCDAWNTFDALIVVGSIVDIAITEVNPAEHTQCSPSMNAEENSRISITFFR    1377
                    ***** : ::..: . :     :*                  . .:: :*.:*** :***

                    ===========~~~~~===4L45=====~========4s5=========~~~~~~~~
Cav1.1              LFRVMRLIKLLSRAEGVRTLLWTFIKSFQALPYVALLIVMLFFIYAVIGMQMFGKIALVD    1296
Cav1.2              LFRVMRLVKLLSRGEGIRTLLWTFIKSFQALPYVALLIVMLFFIYAVIGMQVFGKIALND    1437
                    *******:*****.**:*********************************:****** *

                    ~~~~~~~~~~~~~====4p1=====~~~~==4p2==~~~~~~~~~~~~~~~~~~~~~~~~
Cav1.1              GTQINRNNNFQTFPQAVLLLFRCATGEAWQEILLACSYGKLCDPESDYAP--GEEYTCGT    1354
Cav1.2              TTEINRNNNFQTFPQAVLLLFRCATGEAWQDIMLACMPGKKCAPESEPSNSTEGETPCGS    1497
                     *:*************************:*:***  ** * ***: :     *  **:
```

```
                 =================4s6============~~~~~~~~~=============~~~~~~
Cav1.1           NFAYYYFISFYMLCAFLIINLFVAVIMDNFDYLTRDWSILGPHHLDEFKAIWAEYDPEAK    1414
Cav1.2           SFAVFYFISFYMLCAFLIINLFVAVIMDNFDYLTRDWSILGPHHLDEFKRIWAEYDPEAK    1557
                 .** :******************************************** **********

                 ~~~============~~~~~~~~~~~~=======~~~~~~~~~~~~~~~~~=========
Cav1.1           GRIKHLDVVTLLRRIQPPLGFGKFCPHRVACKRLVGMNMPLNSDGTVTFNATLFALVRTA    1474
Cav1.2           GRIKHLDVVTLLRRIQPPLGFGKLCPHRVACKRLVSMNMPLNSDGTVMFNATLFALVRTA    1617
                 ***********************:***********.*********** ************

                 ~~~~~~~~================~~~~~~~==========
Cav1.1           LKIKTEGNFEQANEELRAIIKKIWKRTSMKLLDQVIPPIGDDEVTVGKFYATFLIQEHFR    1534
Cav1.2           LRIKTEGNLEQANEELRAIIKKIWKRTSMKLLDQVVPPAGDDEVTVGKFYATFLIQEYFR    1677
                 *:******:**************************:** ******************:**
```

Figure S1. Sequence alignment of the Cav1.1 (UniProtKB CAC1S_RABIT) and Cav1.2 (UniProtKB CAC1C_HUMAN) channels. Residues, which are not resolved in the Cryo-EM structure (PDB code 5gjw.pdb) are gray-highlighted. Underlined are some of the residues, which are involved in contacts between VSD-II, AID and Cavβ. Residues G^{402}, G^{406}, and R^{518} whose mutations are associated with TS, are shown in red.

```
β2a       -----------------------------------------MQCCGLVHRRRVRVSYGSA      19
β1c       MVQKTSMSRGPYPPSQEIPMEVFDPSPQGKYSKRKGRFKRSDGSTSSDTTSNSFVRQGSA      60
Cavβ      ------------------------------------------------------------

β2a       DSYTSRPSDSDVSLEEDREAVRREAERQAQAQLEKAKTKPVAFAVRTNVSYSAAHEDDVP      79
β1c       ESYTSRPSDSDVSLEEDREALRKEAERQALAQLEKAKTKPVAFAVRTNVGYNPSPGDEVP      120
Cavβ      ------------------------------------------------------------

β2a       VPGMAISFEAKDFLHVKEKFNNDWWIGRLVKEGCEIGFIPSPVKLENMRLQHEQRAKQGK      139
β1c       VQGVAITFEPKDFLHIKEKYNNDWWIGRLVKEGCEVGFIPSPVKLDSLRLLQEQKLRQNR      180
Cavβ      ------------------------------------------------------------

β2a       FYSSKSGGNSSSSLGDIVPSSRKSTPPSSAIDIDATGLDAEENDIPANHRSPKPSANSVT      199
β1c       LGSSKSGDNSSSSLGDVVTGTRRPTPPASAKQ----------------------------      212
Cavβ      ------------------------------------------------------------

β2a       SPHSKEKRMPFFKKTEHTPPYDVVPSMRPVVLVGPSLKGYEVTDMMQKALFDFLKHRFEG      259
β1c       ----------KQKSTEHVPPYDVVPSMRPIILVGPSLKGYEVTDMMQKALFDFLKHRFDG      262
Cavβ      ----------VVPPAGDVPPYDVVPSMRPIILVGPSLKGYEVTDMMQKALFDFLKHLFDG      307
                        : ..***********::************************* *:*

β2a       RISITRVTADISLAKRSVLNNPSKHAIIERSNTRSSLAEVQSEIERIFELARTLQLVVLD      319
β1c       RISITRVTADISLAKRSVLNNPSKHIIIERSNTRSSLAEVQSEIERIFELARTLQLVALD      322
Cavβ      RISITRVTADISLA-------------------RSSLAEVQSEIERIFELARTLQLVALD      367
          **************                   ************************.**

β2a       ADTINHPAQLSKTSLAPIIVYVKISSPKVLQRLIKSRGKSQAKHLNVQMVAADKLAQCPP      379
β1c       ADTINHPAQLSKTSLAPIIVYIKITSPKVLQRLIKSRGKSQSKHLNVQIAASEKLAQCPP      382
Cavβ      ADTINHPAQLSKTSLAPIIVYIKITSPKVLQRLIKSRGKSQSKHLNVQIAASEKLAQCPP      427
          *********************:***:***************:******:.*::*******

β2a       ELFDVILDENQLEDACEHLADYLEAYWKATHPPSSSLPNPLLSRTLATSSLPLSPTLASN      439
β1c       EMFDIILDENQLEDACEHLAEYLEAYWKATHPPSSTPPNPLLNRTMATAALRRSPAPVSN      442
Cavβ      EMFDIILDENQLEDACEHLAEYLEAYWKATHPPS--------------------------      461
          *:**:***************:*************

β2a       SQGSQGDQRTDRSAPIRSASQAEEEPSVEPVKKSQHRSSSSAPHHNHRSGTSRGLSRQET      499
β1c       LQVQVLTSL-RRNLGFWGGLESSQRGSVVPQEQEHAM-----------------------      478
Cavβ      ------------------------------------------------------------

β2a       FDSETQESRDSAYVEPKEDYSHDHVDHYASHRDHNHRDETHGSSDHRHRESRHRSRDVDR      559
β1c       ------------------------------------------------------------      478
Cavβ      ------------------------------------------------------------

β2a       EQDHNECNKQRSRHKSKDRYCEKDGEVISKKRNEAGEWNRDVYIPQ                    605
β1c       ----------------------------------------------                    478
Cavβ      ----------------------------------------------                    185
```

Supplementary Figure 2. Sequence alignment of Cavβ subunits. Human β1C, GenBank: AAB58779.1; human β2a, GenBank: AAL16948.1. In the rabbit Cav1.1 β-subunit (Cavβ) dashes stand for residues that are not resolved in the cryo-EM structure (PDB code 5gjw.pdb). Residues that interact with the α subunit are highlighted. Among these, only aspartate D307 is not identical (but conserved) between β2a and β1c.

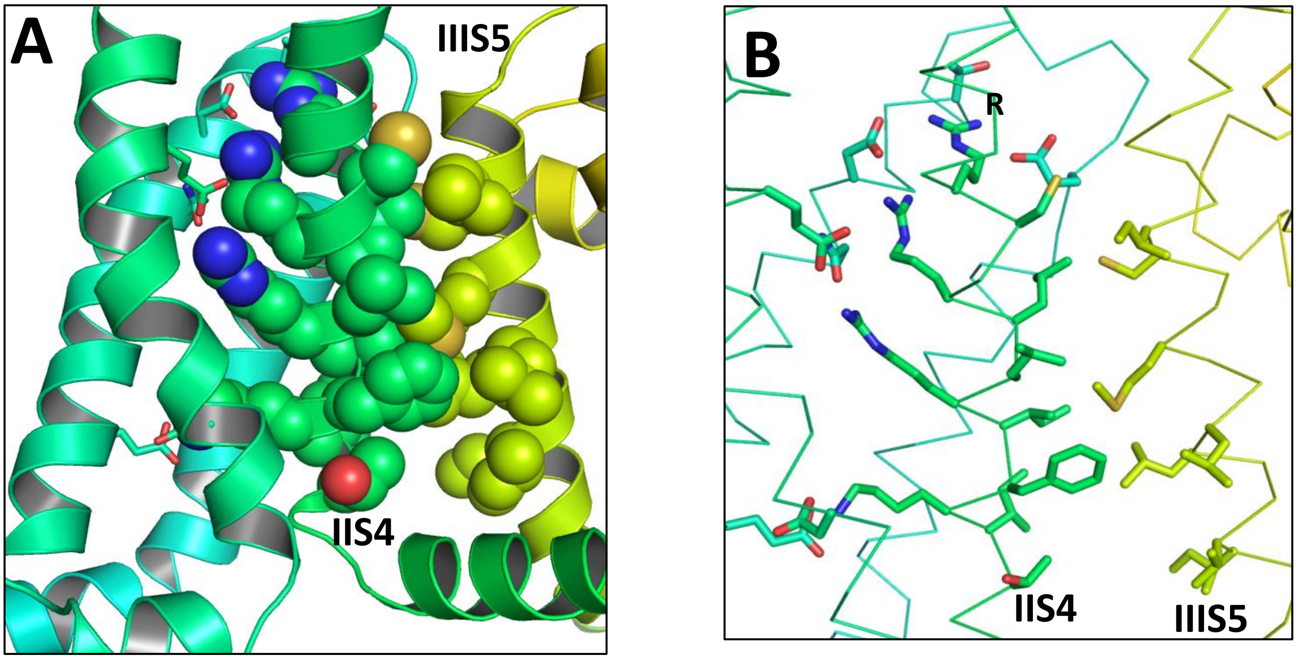

Figure S3. Polar and hydrophobic faces in helix IIS4. Residues in the interface between IIS4 and IIIS5 are shown as spheres (***A***) or sticks (***B***). Hydrophilic face of IIS4 forms predominantly hydrophilic contacts with polar residues in IIS3 and IIS2. The greasy interface between IIS4 and IIIS5 would maintain the interdomain contacts, but minimize friction upon the voltage-dependent shift of IIS4.

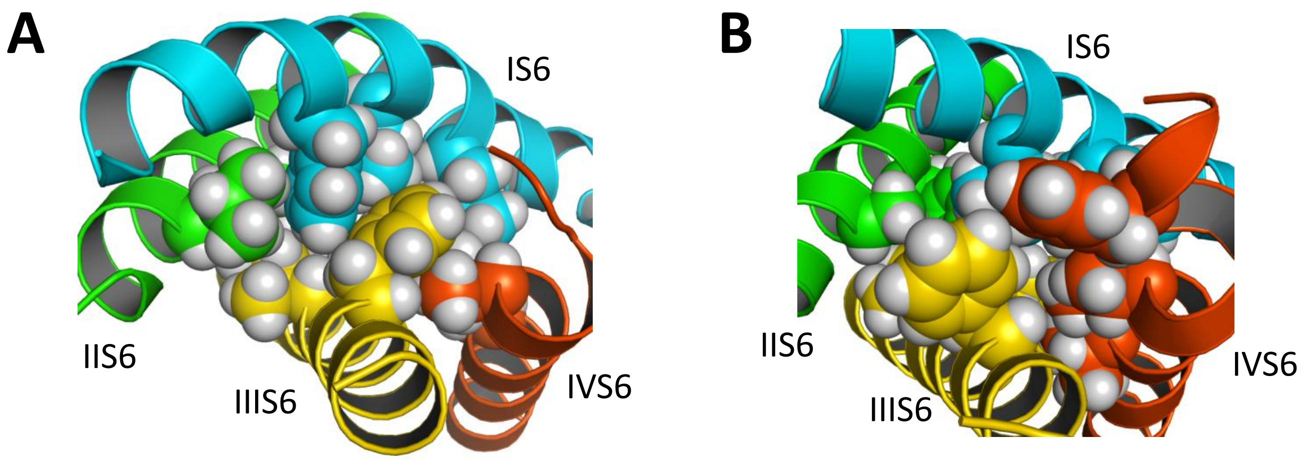

Figure S4. Cytoplasmic views at the C-terminal half of S6 bundle in inactivated-state models ^{i}Cav1.2-Ia (***A***) and ^{i}Cav1.2-II (***B***). The permeation pathway is tightly sealed in both models by several layers of pore-facing hydrophobic residues. This is consistent with the proposition that respective cryo-EM structures capture the channels in non-permeating, likely inactivated state.

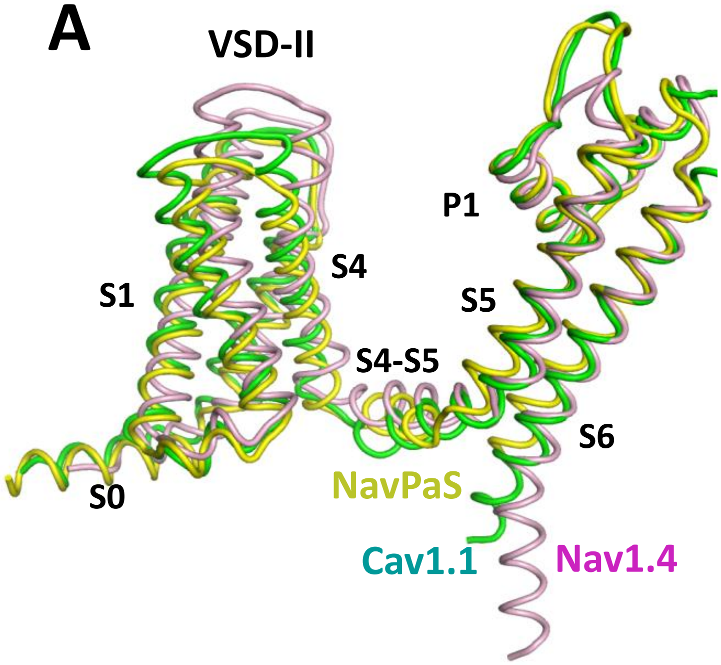

Figure S5. Cav1.1 is more similar to NavPaS and Nav1.4 (A) than to NavAb (B). X-ray structures of Cav1.1 (5gjv), NavPaS (5x0m), EeNav1.4 (5ek0), closed NavAb (5vb2) and open NavAb (5vb8) are 3D aligned by minimizing RMS deviations of C^{α} atoms in the four P1 helices from matching atoms in the reference structure of channel Kv1.2/Kv2,1 (2R9R, not shown). Only repeat II is shown for clarity.

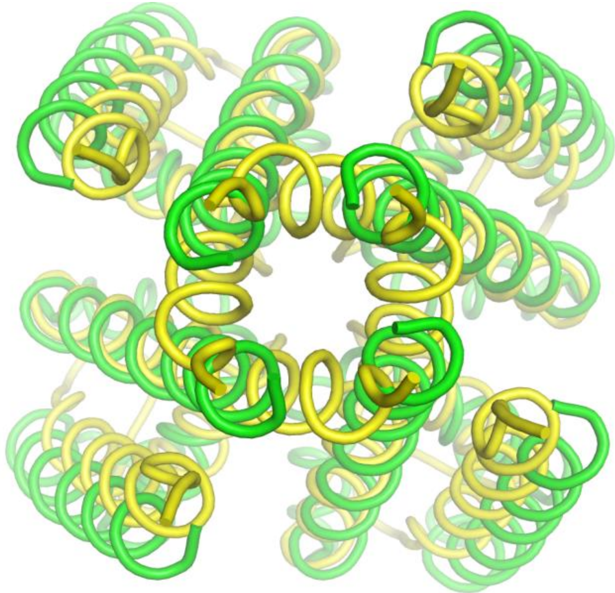

Figure S6. Closed-pore dimensions of NavAb (green, PDB code 5vb2) and KcsA (yellow, PDB code 1bl8) are similar. The structures are superposed by minimizing RMS deviations of C^{α} atoms in the four P1 helices from matching atoms in the reference structure of chimeric potassium channel Kv1.2/Kv2,1 (PDB code 2R9R, not shown).

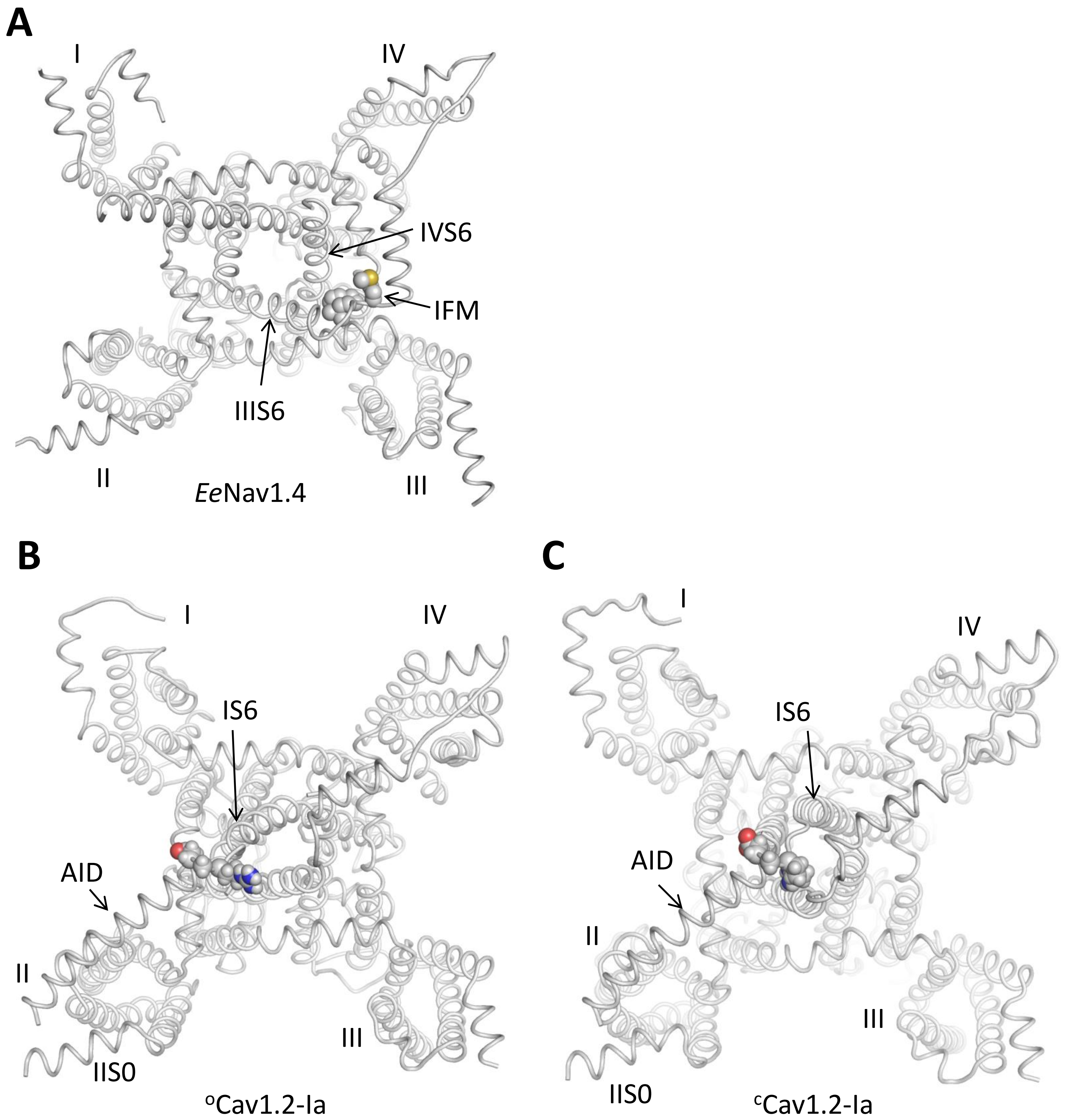

Figure S7. Nav1.4 and Cav1.2 lack "hinged lids". Shown are cytoplasmic views of the channels. ***A*,** In the *Ee*Nav1.4 cryo-EM structure, motif IFM does not block the pore. It would shift IVS6 toward the pore axis, thus initiating the activation gate closure upon fast inactivation. ***B*** and ***C***, N-terminal motif RGD (space-filled) of AID does not occlude the pore in models ^{o}Cav1.2-I (*B*) or ^{c}Cav1.2-I (***C***). Upon membrane depolarization, displacement of IIS0 would push AID towards the pore axis. AID-linked IS6 would bend, initiating the activation gate closure in the process of VDI.
